# tRNA modification profiling reveals epitranscriptome regulatory networks in *Pseudomonas aeruginosa*

**DOI:** 10.1093/nar/gkaf696

**Published:** 2025-07-28

**Authors:** Jingjing Sun, Junzhou Wu, Yifeng Yuan, Leon Fan, Wei Lin Patrina Chua, Yan Han Sharon Ling, Seetharamsing Balamkundu, Dwijapriya, Hazel Suen Suen Chay, Thomas J Begley, Valérie de Crécy-Lagard, Agnieszka Dziergowska, Peter C Dedon

**Affiliations:** Department of Biological Engineering, Massachusetts Institute of Technology, Cambridge, MA 02139, United States; Antimicrobial Resistance Interdisciplinary Research Group, Singapore MIT Alliance for Research and Technology Centre, 138602, Singapore; Department of Biological Engineering, Massachusetts Institute of Technology, Cambridge, MA 02139, United States; Department of Biological Engineering, Massachusetts Institute of Technology, Cambridge, MA 02139, United States; Department of Microbiology and Cell Science, University of Florida, Gainesville, FL 32611, United States; Department of Biological Engineering, Massachusetts Institute of Technology, Cambridge, MA 02139, United States; Antimicrobial Resistance Interdisciplinary Research Group, Singapore MIT Alliance for Research and Technology Centre, 138602, Singapore; Antimicrobial Resistance Interdisciplinary Research Group, Singapore MIT Alliance for Research and Technology Centre, 138602, Singapore; School of Biological Sciences, Nanyang Technological University, 637551, Singapore; Antimicrobial Resistance Interdisciplinary Research Group, Singapore MIT Alliance for Research and Technology Centre, 138602, Singapore; Antimicrobial Resistance Interdisciplinary Research Group, Singapore MIT Alliance for Research and Technology Centre, 138602, Singapore; Department of Biological Sciences and The RNA Institute, College of Arts and Science, University at Albany, SUNY, Albany, NY 12222, United States; Department of Microbiology and Cell Science, University of Florida, Gainesville, FL 32611, United States; Genetic Institute, University of Florida, Gainesville, FL 32611, United States; Institute of Organic Chemistry, Lodz University of Technology, 90-924, Poland; Department of Biological Engineering, Massachusetts Institute of Technology, Cambridge, MA 02139, United States; Antimicrobial Resistance Interdisciplinary Research Group, Singapore MIT Alliance for Research and Technology Centre, 138602, Singapore

## Abstract

Transfer RNA (tRNA) modifications have emerged as critical post-transcriptional regulators of gene expression affecting diverse biological and disease processes. While there is extensive knowledge about the enzymes installing the dozens of post-transcriptional tRNA modifications—the tRNA epitranscriptome—very little is known about how metabolic, signaling, and other networks integrate to regulate tRNA modification levels. Here, we took a comprehensive first step at understanding epitranscriptome regulatory networks by developing a high-throughput tRNA isolation and mass spectrometry-based modification profiling platform and applying it to a *Pseudomonas aeruginosa* transposon insertion mutant library comprising 5746 strains. Analysis of >200,000 tRNA modification data points validated the annotations of predicted tRNA modification genes, uncovered novel tRNA-modifying enzymes, and revealed tRNA modification regulatory networks in *P. aeruginosa*. Platform adaptation for RNA-seq library preparation would complement epitranscriptome studies, while application to human cell and mouse tissue would facilitate biomarker and drug discovery and development.

## Graphical Abstract

**Graphical Abstract:**
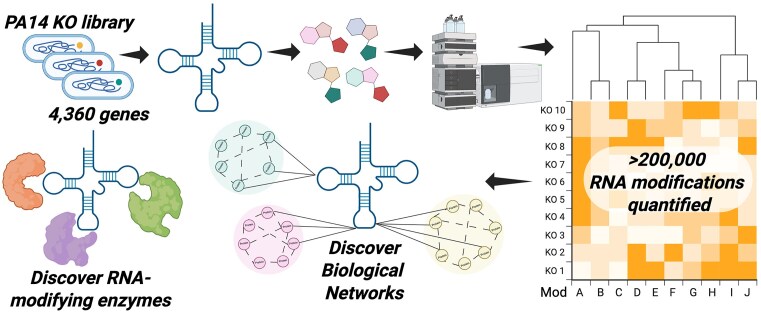

## Introduction

The 170 post-transcriptional RNA modifications comprising the epitranscriptome play a crucial role in regulating mRNA translation in all forms of life. Defects in RNA-modifying enzymes drive dozens of human diseases such as cancer, neurodegeneration, and metabolic disorders [1–3], while RNA modifications also play a role in microbial pathogenesis and antimicrobial resistance [4, 5]. There is growing appreciation for the complexity of mechanisms linking transfer RNA (tRNA) modifications to normal and pathological phenotypes, such as tRNA reprogramming and codon-biased translation in cell stress response and disease [6, 7]. These transcendent behaviors require multi-omic tools to define mechanisms linking upstream environmental sensing and signaling pathways that regulate tRNA-modifying enzymes and the tRNA pool and downstream phenotypic changes in cell physiology and pathology. This systems-level information is critical for validating the dozens of RNA-modifying enzymes as novel drug targets [8–10].

Systems-level analyses of the tRNA epitranscriptome are hindered by a lack of technology for high-throughput (HT) tRNA modification and tRNA pool analysis [11]. This is needed, for example, to screen for tRNA-related translational defects in the 2000 DepMap cancer cell lines [12], screen large microbial gene knockout libraries [13, 14], or whole-cell screening of compound libraries for tRNA-modifying enzyme inhibitors. Major limitations involve artifact-free cell and tissue lysis, cost-effective small and large RNA fractionation, rapid quantification by chromatography-coupled tandem quadrupole mass spectrometry (LC-MS/MS), and lack of software for signal processing and data mining.

**Figure 1.**
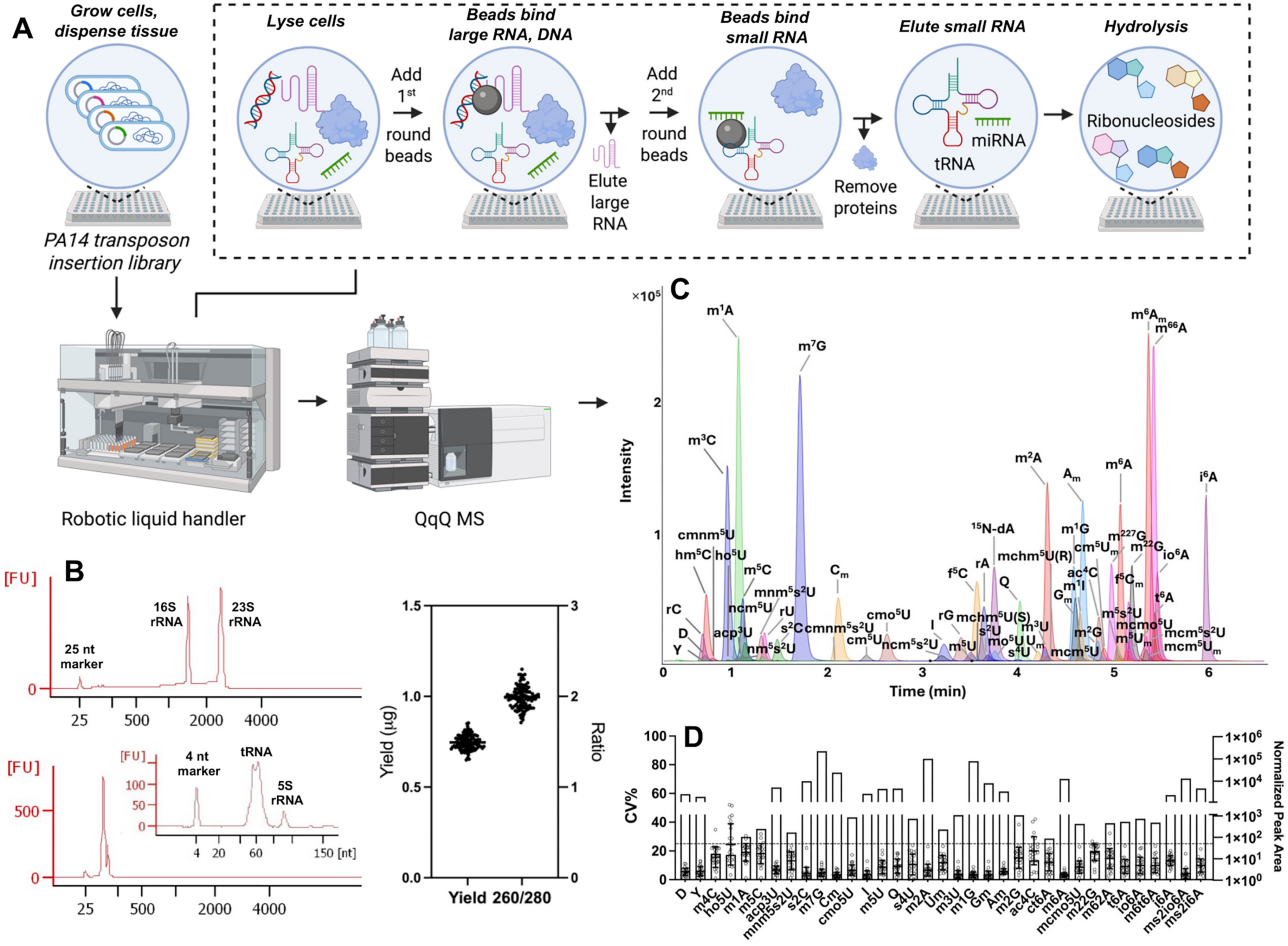
Workflow and validation of a platform for HT tRNA modification profiling of a PA14 mutant library. (**A**) Cell growth, lysis, and small RNA purification in 96-well plate format, with a two-step magnetic beads-based size selection strategy for tRNA purification direct from crude lysate. All steps are performed with a robotic liquid handler (image created at biorender.com and available at available at https://biorender.com/z2jsmlk). (**B**) Characterization of purified RNA fractions from PA14 crude lysate (0.3 OD_600_ cells/well). The Bioanalyzer tracings show the quality of purified large RNAs (upper) and small RNAs (lower) assessed using a “pico chip,” with “small RNA chip” further detailing the RNA quality (inset). The graph shows the small RNA yield of 747 ± 42 ng/well with an *A*_260_/*A*_280_ purity ratio of 1.99 ± 0.12 for *n* = 96. (**C**) The extracted ion chromatogram shows the fast UHPLC-MS/MS method for quantifying 63 modified ribonucleosides (based on synthetic standards) using the dynamic multiple reaction mode (DMRM) of QQQ. (**D**) Quantitative performance of the LC-MS/MS method was evaluated using data from 24 PA14 mutants. The inter-day CV for quantification of 35 RNA modifications was calculated from the average peak area for each RNA modification normalized by the total UV absorbance of the four canonical ribonucleosides (see “Materials and methods” section). The dashed line shows a CV of 25%.

Here we report a robust, HT tRNA modification analysis platform involving bead-based purification of tRNA from cell and tissue lysates, rapid LC-MS/MS analysis of ribonucleosides, and a data processing and mining pipeline (Fig. 1 and Supplementary Fig. S1). The RNA purification leg of the platform was validated with bacteria (*Pseudomonas aeruginosa*), mammalian cells (Hela, HEK293T), and animal tissue (mouse brain). The refined platform were used to screen a *P. aeruginosa* transposon insertion mutant library of 5746 strains covering 4360 nonessential genes [14]. With validation based on loss of known modification genes, the screen revealed hundreds of genes affecting tRNA modification levels and forming regulatory networks for iron-sulfur cluster synthesis and repair, amino acid synthesis, and epitranscriptome enzyme co-factor synthesis, among others. The results provide comprehensive insights into the regulatory landscape of tRNA modifications in *P. aeruginosa*.

## Materials and methods

The experimental and data processing workflows for the HT tRNA modification analysis platform are shown in Fig. 1 and Supplementary Fig. S1, respectively.

### Cell culture and lysis

The 5764-strain nonredundant library of *P. aeruginosa* PA14 transposon insertion mutants [14] was obtained from Dr. Deborah Hung at the Massachusetts General Hospital. Cell culture conditions followed the instructions provided in “The PA14 Non-Redundant Set of *Pseudomonas aeruginosa* Transposon Insertion Mutants User Manual (version 2.2).” A 96-pin replicator was used to inoculate from frozen culture stocks to 0.3 ml of LB medium containing either gentamycin (15 μg/ml) or kanamycin (200 μg/ml) in a 1.2 ml of low profile 96-well plate (BRAND, 701340). The plate was sealed using a breathable film (Sigma, Z763724) and shaken overnight at 37°C, 300 rpm. Then, 100 μl of overnight culture was transferred to a 2.0 ml of deep 96-well plate (VWR, 76329-998) containing 700 μl of LB medium and either gentamycin (15 μg/ml) or kanamycin (200 μg/ml). The plate was sealed and shaking continued at 37°C, 300 rpm, until the cell density reached ∼0.8 OD_600_ (∼1.6 × 10^8^ CFU/ml). Cells were then pelleted by centrifugation of 3000 × *g* for 10 min at 4°C. Medium was removed and cells were washed with cold 1× phosphate-buffered saline (PBS) buffer. After re-pelleting the cells, the PBS was discarded. Cell lysis buffer (100 μl; 50 mM Tris–HCl, 1 mM EDTA, and 4 M guanidine isothiocynate, pH 7.5) was added to each well, and the plate was shaken vigorously at ambient temperature (1500 rpm) for 10 min to ensure complete cell lysis. The quantity of cell lysate was sufficient for two separate RNA isolations.

### RNA purification

The following protocol for RNA purification was used for the robotic workflow detailed below. Large RNA binding was performed by mixing 45 μl of cell lysate with 105 μl of RNA-binding buffer I containing 3 M LiCl, 7% PEG8000, 2 mM EDTA, 40 mM Tris buffer (pH 7.5), and 8 μl of Sera-Mag carboxylate-modified magnetic beads (Cytiva, 65152105050350; “first round”). Under these conditions, large RNAs, but not small RNAs, bind to the first round carboxylate-coated magnetic beads. The mixture was agitated thoroughly and incubated at ambient temperature for 5 min. The magnetic beads were separated on a magnetic rack at ambient temperature for 5 min, and the supernatant containing small RNAs and proteins was transferred to a new tube/96-well plate. The tube/plate was then centrifuged at 3000 × *g* for 1 min and placed on the magnetic rack for 5 min to remove any remaining beads. The supernatant was transferred to a new tube/plate and mixed with 1.8× (v/v) RNA-binding buffer II (0.05% magnetic beads in isopropanol; “second round”). Under these conditions, small RNAs bind to the second round carboxylate-coated magnetic beads. The mixture was agitated thoroughly and incubated at ambient temperature for 5 min. The magnetic beads were separated on the magnetic rack for 5 min, and the supernatant was removed. The pelleted beads from first round and second round binding reactions were washed twice using washing buffer (10 mM Tris–HCl, 80% EtOH, pH 7.5). The supernatant was removed and the beads were air dried at ambient temperature. Large RNAs (>150 nt), including 16S and 23S ribosomal RNAs (rRNAs) and some mRNAs, were eluted in 50 μl of nuclease-free water at 90°C for 2 min. Small RNAs (<150 nt; ∼85% tRNA, 5S rRNA, rRNA/mRNA fragments) were eluted in 50 μl of nuclease-free water at ambient temperature.

### Robotic workflow for RNA isolation

A robotic liquid handler (Tecan EV150, Switzerland) was used to perform the magnetic beads-based RNA isolation in 96-well plate format. The layout of the worktable is shown in Supplementary Fig. S2 and the operating parameters detailed in Supplementary Table S1. *Large RNA binding (first round beads)*: The plated cell lysates were placed on ice to thaw while reagents were prepared in 96-well plates for RNA isolation. The plate containing thawed cell lysates (45 μl) was placed in deck position 1. A 96-chanel pipette (MCA96) was used to transfer cell lysates to plate containing RNA binding buffer-1 (105 μl, deck position 5) and mix the reagents with five mix cycles. The plate then was incubated for 5 min to allow large RNA binding to the magnetic beads. *Wash buffer aliquot*: During this period, the wash buffer was being aliquoted (130 μl × 5) from trough “Wash Buffer” to plate “LRNA WB” (deck position 4). *Beads pulldown (1)*: The plate then was transferred by the robotic manipulator arm (RoMa) to magnetic plate (Alpaqua, Catalyst 96, A000550) “Magnet1” (deck position 10) for magnetic beads pulldown. *Wash buffer aliquot*: During the awaiting time, the wash buffer was being aliquoted (130 μl × 5) from trough “Wash Buffer” to plate “tRNA WB” (deck position 12). *Beads pulldown (2)*: The supernatant (145 μl) was then transferred to plate “Supernatant1” (deck position 6), and the residue was removed and discarded in waste plate “LRNA Waste” at deck position 8. Upon completion, the program was suspended, the plate “Supernatant1” was manually removed and placed in a centrifuge (3000 × *g*, 1 min) to spin down remaining first round beads. The plate was placed back on magnetic plate “Magnet2” (deck position 9) and the program continued. *Beads wash*: During the waiting time for the first round beads residue pulldown on “Magnet 2,” the first round beads in plate “LRNA MB” on “Magnet1” (deck position 10) were washed with an aliquot of wash buffer from “LRNA WB” (300 μl). The wash buffer residue was removed and discarded after 60 s. After repeating the wash step, the first round beads were left to air dry. *tRNA binding (second round beads)*: During this period, supernatant (120 μl) in plate “Supernant1” on “Magnet2” was transferred to plate “tRNA MB” (deck position 7) and mixed thoroughly with RNA-binding buffer II (228 μl). *Large RNA elution*: While the tRNA was binding to the magnetic beads, nuclease-free water (55 μl) in a trough was added to the air-dried first round beads in plate “LRNA MB.” The plate was manually removed and shaken on an orbital plate shaker (1000 rpm, 60 s), then incubated in a metal beads bath (90°C, 90 s). Then the plate was placed back on “Magnet2” for 20 s for pulldown of the beads. The elutes (50 μl) were transferred to plate “LRNA Elute” (deck position 2), the plate was placed on ice immediately. *Beads pulldown*: The plate “tRNA MB” was transferred to “Magnet1” to pull down the beads. The supernatant was removed through three separate dispensing actions, with a 60 s interval between each to allow sufficient time for the magnetic beads to fully settle. This stepwise approach is essential due to the significant distance between the magnet plate and the beads, preventing incomplete bead recovery in a single dispensing action with substantial bead loss. *Beads wash*: The second round beads were washed by aliquoted wash buffer from “tRNA WB” (300 μl). The wash buffer residue was removed and discarded after 60 s, then the second round beads were left air dry. While awaiting the beads airdry, the preparation for next run can be performed. *tRNA elution*: Nuclease-free water (55 μl) in trough was added to airdried second round beads in plate “tRNA MB” on “Magnet1.” The plate was manually taken out and shaken on an orbital plate shaker (1000 rpm, 60 s). The plate was put back on “Magnet1” and wait 20 s for beads pulldown. Upon the elutes (50 μl) were transferred to plate “tRNA Elute” (deck position 3), the plate was kept on ice immediately until transferred for LC-MS sample preparation or stored at −80 °C for long-term storage.

### RNA hydrolysis for LC-MS/MS analysis

The purified RNAs in the 96-well PCR plate (Axygen Scientific, PCR-96-FS-C) were transferred to a 0.3 ml of 96-well plate (Agilent, 5043-9313) for hydrolysis in a 40 μl of an enzyme cocktail containing 10 U benzonase (Sigma, E8263), 4 U calf intestinal alkaline phosphatase (Sigma, 524572), 0.1 U phosphodiesterase I (US Biological, P4072), 0.1 mM deferoxamine (Sigma, D9533-1G), 0.1 mM butylated hydroxytoluene (Sigma, W218405), 4 ng coformycin (NCI, 27 781 713), 50 nM internal standard [^15^N]_5_-2'-deoxyadenosine, 2.5 mM MgCl_2_, and 5 mM Tris–HCl buffer pH 8.0. The reaction mixture was incubated at 37°C for 6 h.

### Liquid chromatography-coupled tandem mass spectrometry

#### Dynamic MRM scans

For RNA modification retention time (RT) validation (Supplementary Table S2), synthetic standards were utilized in tandem with a Waters ACQUITY UPLC BEH C18 column (50 × 2.1 mm, 1.7 μm) equipped with an 0.2 μm stainless steel inline filter (Waters, 205 000 343) connected to an Agilent 1290 Infinity II UHPLC system and an Agilent 6495 triple-quadrupole mass spectrometer. The LC was operated at 25°C with a flow rate of 0.35 ml/min. Initial conditions held 100% solution A (water, 0.02% formic acid) for 2 min, followed by 2–4 min at 0%–8% solution B (70% acetonitrile, 0.02% formic acid), and from 4 to 5.9 min at 8%–100% solution B. The mass spectrometer was operated in positive mode with an electrospray ionization source with following parameters: gas temperature of 200°C, gas flow of 11 L/min, and a capillary voltage of 3000 V. Detection leveraged a dynamic MRM mode, targeting product ions from precursor ions of each RNA modification. The collision energy (CE) was optimized by MassHunter Optimizer for maximal sensitivity for the modification. The hydrolysed RNAs in the 96-well plate were sealed with easy piercing film (BioChromato, REPS001, Japan) and kept at 5°C during analysis.

#### Neutral loss scan

The MS was operated in positive ion mode, using Agilent MassHunter software in a neutral loss scan (NLS) setting. At collision energies of 10 and 20 eV, the neutral losses of 132 (for ribose) and 146 (for methyl-ribose) were monitored within a mass range of 200–500 Da. The NLS analyses used 5 μg of hydrolysed tRNA.

Note: We observed that use centrifugal filtration devices to remove enzymes and proteins prior to LC-MS analysis biased the analysis toward nonpolar RNA modifications. We thus avoided use of these devices. While use of a 2-μm inline filter between the injector and the LC column protects against particulate contamination, the enzymes still enter the LC column and risk performance degradation. However, such degradation was not apparent in our analysis.

### LC-MS data processing and analysis

As illustrated in Supplementary Fig. S1, the UV and LC-MS/MS raw data are batch processed (*n* = 96, by plate) using Masshunter Qualitative Analysis (Agilent, Version 8.0) and Masshunter Quantitative Analysis (Agilent, Quant-My-Way version 10.1) separately, and transformed to .csv files. If not otherwise specified, MS data possessing method employs following parameter: Agile2 integrator algorithm, peak filter of 300 counts, left/right RT Delta 1 min, noise algorithm peak-to-peak, noise SD multiplier of 5 min, S/N 5, Accuracy Max 20% max %Dev, and smoothing function is off. For peaks with distortions (cmo^5^U, ho^5^U) and for co-eluting isobaric isomers (m^4^C/m^5^C, m^1^G/m^2^G, I/A, and ho^5^U/s^2^C), the left/right RT Delta is reduced according to the RT difference. The output data from Masshunter underwent an immediate review using R to detect any shifts in RT. The script identified instances where the RT exceeded the mean value for the plate by >0.2 min. These cases were then flagged for a manual assessment in Masshunter to verify the precision of the peak selection process. This step is crucial for ensuring the reliability of the data before proceeding with further analysis.

An R script developed for data processing iterates through each MS data file in the directory, performing a series of steps on the data: (i) *Data cleanup and extraction*: For each file, it extracts MS and UV signals, and identifies samples with UV variation >80% of the mean of each 96-sample. These samples were subjected to manual review and subsequently excluded from further analysis to prevent bias in LC-MS results due to excessively divergent sample input. (ii) *Normalization and fold-change calculation*: The raw MS peak area of each modification (rM) is normalized by the sum of canonical ribonucleosides (rN) UV signals $ {{\mathrm{Normalized\ rM\ signal\ (rMi) = }}\frac{{rM{\mathrm{ }}\ raw\ signals}}{{\sum {UV\ singals\ of\ canonical\ rNs} }}}$ in each experiment as a control for equal sample loading into the instrument. Note that the normalized MS data do not reflect absolute abundance and cannot be compared directly between experiments run at different times. A list of modifications existing in the WT strain was subsequently processed to calculate fold-change values. To compensate for signal drift over the analysis time course of each 96-well plate, the fold-change was calculated in a row-based manner that was analyzed at 2 h using the following equation: $( {{\mathrm{Fold - change }}({\mathrm{FCi}}) = \frac{{rMi }}{{{\mathrm{Row\ means\ of\ rMi}}}}} )$. To minimize the impact of extreme values, any values >200% or <50% of the initial row means were omitted before recalculating the final row means, which then form a baseline for fold-change computation for all samples in this row. (iii) *Annotation and data output*: The normalized peak area and fold-change results were cross-referenced with mutant details (such as Gene Name, Gene ID, etc.) and saved as CSV files for further analysis. (iv) *Data filtering and evaluation*: Mutants displaying substantial fold-changes in any modification (>2 or <0.5) have been identified for subsequent network analyses. Furthermore, mutants with >10 modifications exhibiting a fold-change >1.5 or <0.7 were manually reviewed to preclude false positives in biological findings, potentially introduced by LC-MS artifacts that could result in an apparent overall upregulation or downregulation of RNA modifications.

### Arbitrary PCR and DNA sequencing

Specific PA14 mutants from frozen glycerol stocks was cultured on LB agar plates using a quadrant streaking method. These plates were incubated at 37°C overnight. For each mutant, two single colonies were picked into 3 ml of LB medium (either 15 μg/ml gentamycin or 200 μg/ml kanamycin) in 15 ml of falcon tube and grown at 37°C overnight (300 rpm). The overnight culture was transferred into a new 1.5 ml of tube and stored at −20°C for 1 h. The tubes were thawed and incubated at 99°C for 10 min to lyse the cells. The cell lysate was homogenized by pipet up and down and spun at 3500 rpm for 5 min to pellet cell debris. Two-step arbitrary PCR was performed by following the instructions in the website for the “PA14 Transposon Insertion Mutant Library” [14] (https://pa14.mgh.harvard.edu/cgi-bin/pa14/home.cgi). The PCR products were sent for Sanger sequencing (Axil Scientific, Singapore), using PMFLGM.GB-4a (5′-GACCGAGATAGGGTTGAGTG-3′) as the sequencing primer.

### Bioinformatics

Database and tools including Modomics [15], Uniprot[16], BV-BRC [17], NCBI [18], HHpred [19], and PA14 mutant library [14] (https://pa14.mgh.harvard.edu/cgi-bin/pa14/home.cgi) were routinely used for bioinformatic analyses. Modification and protein network were produced in Cytoscape v3.10 [20]. Heatmap and clustering were performed using package “ComplexHeatmap” [21] in R. Protein–protein interaction network was produced using STRING database [22], with setting “interaction sources: experiments, co-expression, neighborhood, gene fusion, and co-occurrence; minimal confidence score: 0.5; interactors: query protein only.” SAPPHIRE.CNN [23] and BPROM [24] were used for transcription start site and promoter prediction. Protein IDs used in this study are listed in Supplementary Table S3.

### Multiple sequence alignment and structure alignment

Sequence alignment was performed using MUSCLE [25] and viewed in Jalview [26]. Amino acids were colored according to physicochemical properties. The structure of PA14_14340, PA14 RlmN, PA14_68100, PA14_40730, PA14_16930, PA14_17650, TapT_Ec_, and PA14_51680 were generated by SWISS-MODEL [27] and Alphafold [28]. The protein structure models were then subjected to NPDock (Nucleic acid-Protein Dock) [29] using the default setting with the structure of *Escherichia coli* tRNA^Asp^ (PDB: 6UGG), tRNA^Glu^ (PDB: 5HR6), and tRNA^Val^ (PDB: 7EQJ) for the prediction of the protein–RNA complex structure. Briefly, a low-resolution method GRAMM was used to generate 20 000 alternative models (decoys) with physically reasonable geometric compatibility between protein and RNA structures. Then, the decoys were scored and clustered according to their mutual similarity, to retain groups of very similar decoys. The overall best-scored complex, as well as a representative of the largest cluster of well-scored decoys, was selected to present. Structure was analyzed and visualized using PyMol (version 2.5).

### Phylogenetic analysis

For the tree of representative bacteria, a maximum likelihood tree of 10 concatenated ribosomal proteins was generated as previously described [30]. For the tree of RlmN and PA14_40730, sequences of 5965 RlmN and 136 PA14_40730 proteins were obtained by BLASTp search (cutoff: percentage of identity 30%; *E*-value 1e^−20^) in 6616 representative bacterial genomes in BV-BRC database as collected in January 2021. METTL3 proteins in human, mouse, and *Drosophila* were used as the out group. For the tree of PA14_68100, sequences of 1460 TrmH and 340 PA14_68100 proteins were obtained by BLASTp search. The obtained protein sequences were aligned using MUSCLE [25] and trimmed by BMGE v1.12 [31]. The tree is inferred using FastTree [32] with default parameters and 100 bootstrap replicates generated with SeqBoot [33]. The trees are visualized using iTOL [34]. Branches are colored by phylogenetic affiliation at phylum level.

## Results

### Developing a high-throughput platform for epitranscriptome profiling

The platform workflow is shown in Fig. 1A: (i) cells or tissue samples in wells of a 96-well plate are lysed, (ii) large RNA and genomic DNA are removed and stored for future study, (iii) small RNA is isolated, and (iv) hydrolyzed to ribonucleosides for (v) analysis by LC-MS/MS. Each step was optimized as detailed in Supplementary Results and Supplementary Figs S2–S7. While 4 M guanidine isothiocyanate (GITC) was sufficient to lyse bacteria and human cells, animal tissues required some form of mechanical disruption (e.g. tissue lyser; Supplementary Fig. S3). Both approaches inhibited RNases and RNA-modifying enzymes and provided high quality RNA for size-based resolution of large and small RNAs using a two-step beads-based method (Fig. 1A and Supplementary Fig. S3). The first step removes genomic DNA and large RNAs (>150 nt, mainly rRNA and mRNA, including 16S and 23S rRNA for prokaryotes and 5.8S, 18S, and 28S rRNA for eukaryotes), with small RNAs (<150 nt, ∼85% tRNA, 5S rRNA, miRNA, and rRNA/mRNA fragments) captured in the second step. Both silica- and carboxyl-coated magnetic beads were effective for bacterial and mammalian cells, but carboxyl-coated beads were chosen due to compatibility with tissues (Supplementary Fig. S3F). The method was as effective as commercial kits for size resolution and tRNA yield (Supplementary Fig. S3E).

Cell lysis and tRNA purification were then adapted to 96-well plates for an automated, 1-h, 9-step workflow on a robotic liquid handler (Tecan EVO150) (Supplementary Fig. S2A and B), with empirical optimization of labware and liquid handler parameters (Supplementary Table S1) for consistent results (Fig. 1B; Supplementary Figs S2C, D, and S7). This approach provided an average yield of small RNA from 0.3 OD_600_ of *Pseudomonas* cells is 747 ± 42 ng (*n* = 96) with an average *A*_260_/*A*_280_ ratio of 1.99 ± 0.12 (Fig. 1B). The method produced high-quality tRNA at low cost ($0.3/sample) and high efficiency (1 h for 96 samples).

The LC-MS/MS RNA modification analysis required optimization of RNA hydrolysis, HPLC resolution, and MS/MS quantification of ribonucleosides to maximize sensitivity and specificity and to minimize run time. Using a published RNA hydrolysis protocol [35], we determined that an adenosine deaminase inhibitor, here coformycin, was essential for accurate inosine levels, but a cytidine deaminase inhibitor was unnecessary as no significant m^3^C/m^5^C to m^3^U/m^5^U deamination was detected (Supplementary Fig. S6A). Further, the iron chelator deferoxamine was used to prevent ho^5^U oxidation [35]. Lastly, particulate removal was achieved using a 0.2 μm stainless steel inline filter rather than a polyethersulfone 10K spin-filter to prevent significant loss of hydrophobic ribonucleosides (Supplementary Fig. S6B and C). For chromatographic resolution of ribonucleosides, we chose a rapid UHPLC method using a BEH C18 column with dynamic multiple reaction monitoring for analysis of >60 RNA modifications in a 6-min HPLC run (Fig. 1A). This proved useful for bacteria, mammalian cells, and animal tissues (Fig. 1C and Supplementary Figs S3–6). Both the LC configurations (Supplementary Fig. S4) and MS parameters (Supplementary Table S2) were optimized for identifying (e.g. isobaric methylations, Supplementary Fig. S4; problematic U modifications, Supplementary Table S4) and quantifying modifications (low femtomole limits of detection and quantification, Supplementary Table S4). Using tRNA from wild-type PA14, the standard deviation for HPLC RTs was <5 s (Supplementary Fig. S5A), and the coefficient of variation (CV) for peak area <10% (Supplementary Fig. S5B and C), with negligible sample carryover (Supplementary Fig. S5D and E). To validate LC-MS/MS method reliability, we analyzed the same sample matrix (*n* = 16) using both fast (6 min) and long (30 min) methods and found a strong correlation (Supplementary Fig. S5F; Pearson correlation, *r* = 0.927, *P* < 0.0001).

The final platform functionality is a data processing pipeline (Supplementary Fig. S1, “Materials and methods” section) to (i) convert hardware-specific signals to normalized signal intensities comparable across analytical runs, (ii) collate signal intensities with gene names, and (iii) calculate fold-change values. The lack of antibiotic resistance in wild-type strain prevented its growth in the medium used for mutants, obviating its use as a control for fold-change calculations. Here we exploited the fact that most mutants exhibited insignificant changes in most modifications, with >94% of 172 860 measurements showing <1.2-fold changes relative to an adjusted mean of all samples run in the same 2 h window. The adjusted mean value for each modification was calculated by averaging normalized peak areas (relative to UV absorbance of canonical ribonucleosides), eliminating data for samples changing >2-fold, and calculating the adjusted mean. This approach also protected against LC-MS signal drift over the course of a day-long run.

The complete platform processed a 96-well plate for tRNA purification in 1 h followed 15 h for 96 LC-MS/MS analyses (9.4 min/sample). We next applied the platform to a 5746-strain PA14 gene knockout library.

### tRNA modification profiling of a PA14 transposon insertion mutant library

To identify genes affecting tRNA modifications and thus discover epitranscriptome regulatory mechanisms, we applied the tRNA modification analytical platform to the nonredundant *P. aeruginosa* UCBPP-PA14 transposon insertion mutant library consisting of 5746 mutant strains covering 4600 nonessential genes [14]. This required identifying and reproducibly quantifying PA14 tRNA modifications. Here we created a dataset from modifications reported for wild-type PA14 [36, 37] and for Gram-negative *Escherichia coli* [38] (Supplementary Table S5). Under our bacterial culture conditions (“Materials and methods” section), we identified 35 modifications in wild-type PA14 tRNA, with an additional six (cmnm^5^s^2^U, nm^5^s^2^U, mo^5^U, preQ_1_, oQ, and s^2^U) detected as biosynthetic intermediates in specific PA14 mutants. Of these 41, 37 were validated with synthetic standards, while m^6^t^6^A and ms^2^io^6^A were identified by neutral loss analysis and high-resolution mass spectrometry (Supplementary Fig. S8) [38]. ct^6^A and oQ were identified by quantifier and qualifier MS transitions and their absence apparent in mutant strains lacking known synthetic enzymes (ΔTcdA and ΔQueA, respectively). Platform performance for quantifying the 41 modifications was assessed with 24 PA14 mutant strains (positioned in the first three columns of Plate 1) in four biological replicates. While most modifications had a CV < 25% (Fig. 1D), five exhibited high variation in signal intensity (ac^4^C [39], ho^5^U [40], m^1^A [41], m^4^C [42], and m^2,2^G [43]), with measurement deemed unreliable due to recognized chemical instability [44], low abundance, or possible absence in PA14.

**Figure 2.**
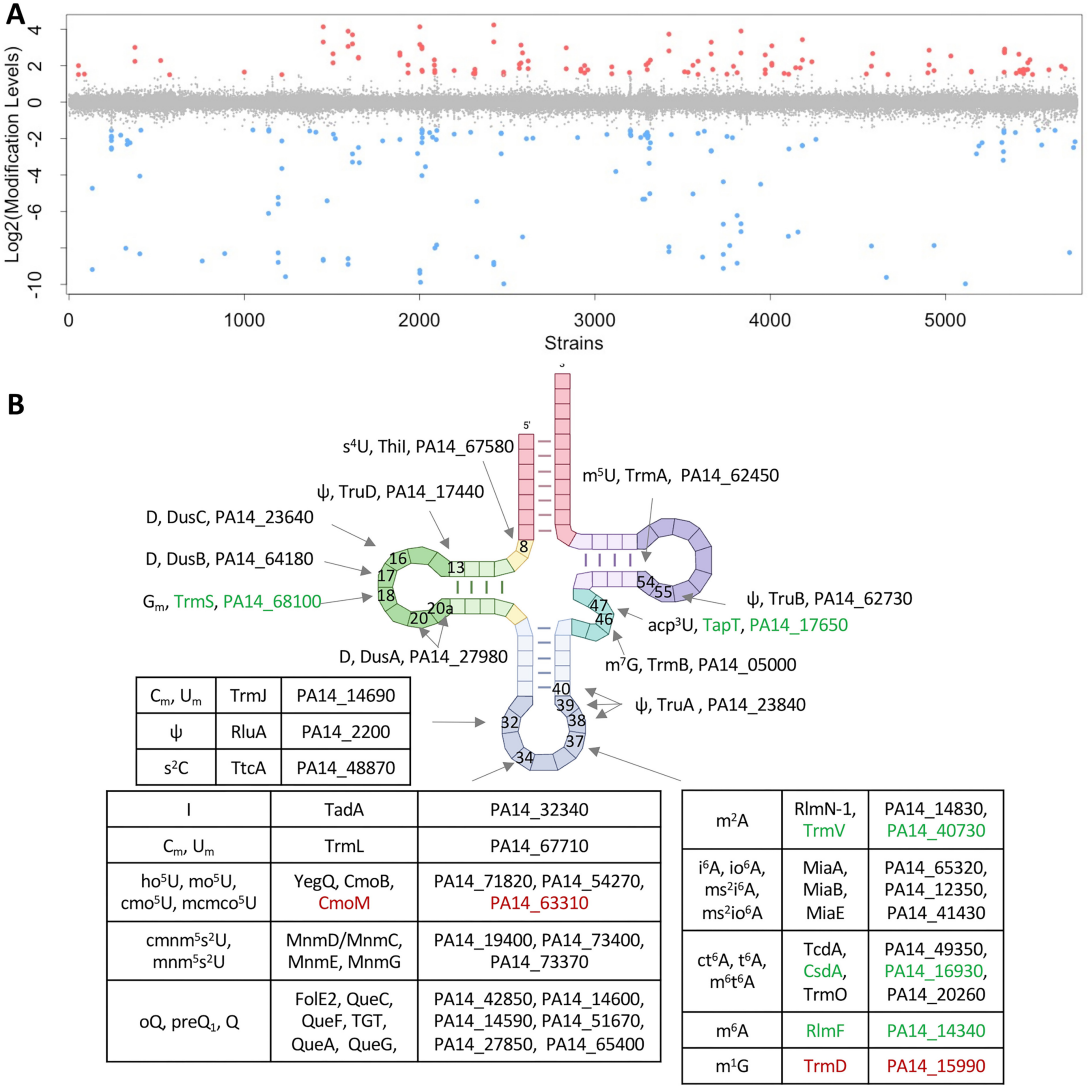
tRNA modification profiling in the PA14 mutant library reveals a constellation of gene-modification linkages. (**A**) Genes regulating the levels of tRNA modifications are visualized in this scatter plot of RNA modification fold-change values (calculated as described in “Materials and methods” section, *y*-axis) across 5746 strains in the PA14 gene knockout library (*x*-axis). Modifications with log_2_(fold-change) > +1 are noted with red circles, < -1 in blue, and between -1 and +1 in gray. (**B**) PA14 tRNA modifications and corresponding modifying enzymes validated and identified in this study. The unannotated proteins in green are newly identified as tRNA-modifying enzymes in this study. The mutants in red were confirmed to be absent in the library by both LC-MS and sequencing results. The four-leaf clover tRNA was created at biorender.com, available at https://biorender.com/pxfpddu.

The validated platform with 35 RNA modifications was now applied to the complete PA14 library. The entire screen required ∼60 h of sample processing and ∼900 h of LC-MS/MS, generating > 200 000 RNA modification signals. LC-MS/MS data were processed according to the flowchart in Supplementary Fig. S1, and the results are presented in Supplementary Table S6. As shown in Fig. 2A, most of the modifications did not change by >2-fold relative to their adjusted mean (gray points in Fig. 2A). However, the loss of 313 genes covered by 352 mutant strains caused 30 modifications to change significantly (>2-fold; red and blue points in Fig. 2A; Supplementary Table S6C). Manual curation of the dataset revealed several misannotated strains and strains with adventitious transposon insertion sites causing tRNA modification patterns inconsistent with expectation. These discrepancies, potentially arising from insertions near the N-terminal/C-terminal encoding region or polar effects impacting adjacent gene expression, were checked by polymerase chain reaction (PCR), reverse-transcription PCR (RT-PCR), genome sequencing, and/or transcriptional analysis. For example, intragenic insertions closed to N-terminal could still allow functional AroB and QueE proteins (Supplementary Fig. S9 and Supplementary Results). With few exceptions, the fidelity of most mutants was high, a conclusion supported by the strong correlations between modification levels and loss of known or suspected RNA-modifying enzymes.

### Confirming and discovering RNA modifying activities

**Figure 3.**
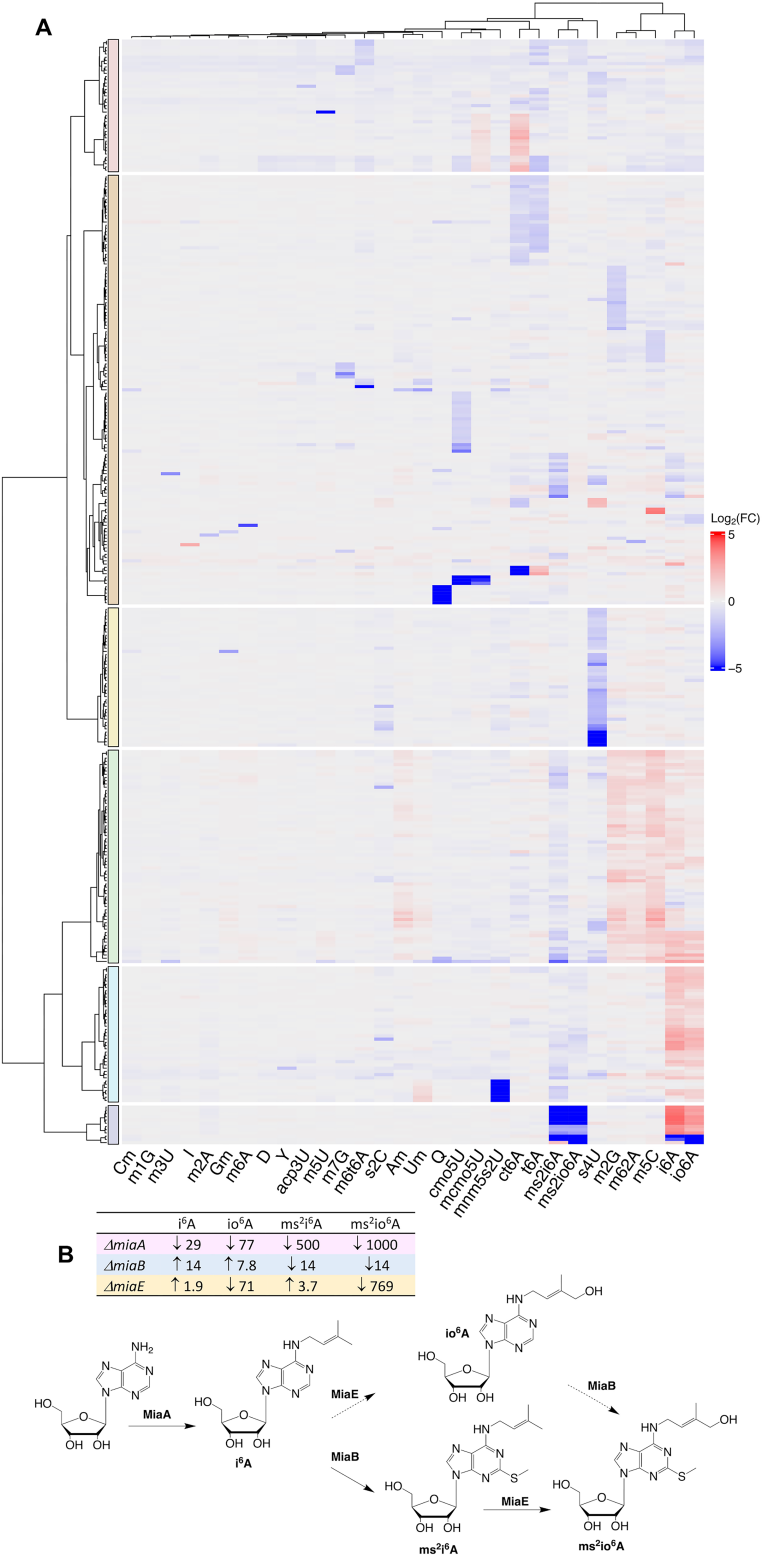
Cluster analysis reveals gene families affecting the tRNA epitranscriptome. (**A**) Hierarchical clustering of fold-change values for 30 tRNA modifications for the 352 PA14 mutants (including duplicates) possessing significantly altered modification levels. The fold-change calculation is described in **Methods**. (**B**) Biosynthetic pathways for i^6^A-derived tRNA modifications. The inset table details the fold-change increase or decrease in the levels of modified ribonucleosides in the mutant strains lacking synthetic enzymes. Dashed lines represent proposed enzyme activities based on results from the PA14 screen. Data are derived from Supplementary Tables S6 and S7.

Screening of the 5746 PA14 mutants yielded a rich dataset of 313 genes significantly affecting 30 tRNA modifications. As an invitation to readers to explore these data futher, we illustrate mining of this dataset at several levels, starting with hierarchical clustering to identify co-variance among mutant strains (Fig. 3A). This analysis revealed several clear interdependencies among modifications, as well as novel and unannotated gene function and subtle enzyme–substrate relationships. At the simplest level, loss of 30 genes encoding enzymes known to synthesize RNA modifications in PA14 (Fig. 2B and Supplementary Table S7) caused an absence or a decrease (for redundant writers) in the modification levels (Fig. 3A; Supplementary Tables S5–S7). The analysis also revealed clusters of genes differentially altering levels of several groups of modifications, such as such as the i^6^A family and t^6^A and ct^6^A. This was particularly notable for the position 37 i^6^A family: io^6^A, ms^2^i^6^A, and ms^2^io^6^A (Fig. 3B). As expected, the levels of these modifications all decreased with loss of *PA14_65 320*, *miaA* (pink box, top row in Fig. 3B), the enzyme catalyzing isopentyl transfer from dimethylallyl diphosphate to N^6^-A in tRNA to form i^6^A37 (Fig. 3B), the precursor to the others. MiaB inserts a sulfur in i^6^A to form ms^2^i^6^A [45] and its loss in two mutant strains increased i^6^A and io^6^A by an average of 14- and 7.8-fold, respectively, and reduced ms^2^i^6^A and ms^2^io^6^A by 14-fold (Fig. 3B). The observed increase in io^6^A levels by 6- to 9-fold in the two *miaB* mutant library replicates (Supplementary Table S6) suggest that io^6^A is a substrate for thiolation by MiaB to ms^2^io^6^A (dotted line, Fig. 3B), though this needs direct confirmation. In other bacteria, MiaE oxidizes a side-chain carbon in i^6^A and ms^2^i^6^A [46]. Here loss of PA14_41430 results in increased levels of these modifications and reduced levels of the oxidation products io^6^A and ms^2^io^6^A (Fig. 3B), which provides functional annotation for this gene product as MiaE in PA14. In terms of more subtle substrate selectivity, our results also support the observed preference of MiaE for ms^2^i^6^A over i^6^A [46, 47]: in wild-type the ms^2^io^6^A abundance is ∼40 times higher than io^6^A and the io^6^A/i^6^A ratio is ∼0.2, while the ms^2^io^6^A/ms^2^i^6^A ratio is ∼4 (Fig. 3B). These results are consistent with known and new substrate–product relationships for the position 37 i^6^A modifications and their catalytic enzymes. Importantly, the PA14 library screen revealed a broad network of 49 genes causing i^6^A and io^6^A levels to be negatively correlated with ms^2^i^6^A and ms^2^io^6^A (Fig. 3A and Supplementary Table S6), as discussed shortly.

**Figure 4.**
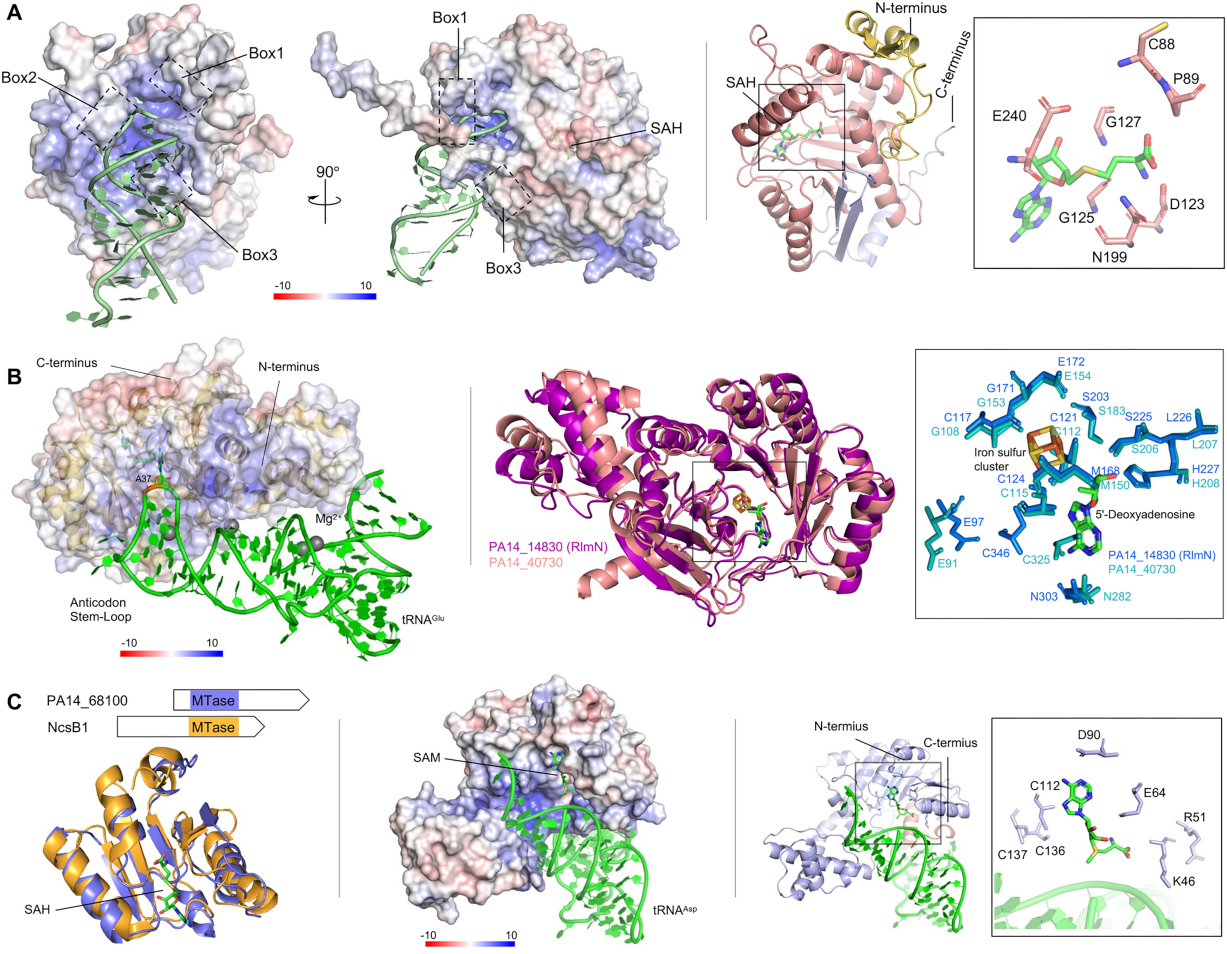
Structure modeling of RlmF, TrmV, and TrmS. (**A**) Left: Putative tRNA-binding site of RlmF (Alphafold model). The ASL of tRNA^Val^ is represented by green cartoon. Three helix-turns that surround the groove of ASL are boxed. Right: the structure modeling of RlmF. The sticks boxed represent the key residues in the proximity of SAH. (**B**) Left: Putative tRNA-binding site of TrmV (Alphafold model). The tRNA^Glu^ is represented by green cartoon with A37 shown in stick format. Right: The structure alignment of PA14 RlmN and TrmV. The stick boxed represent the key residues in the catalytic pockets of the two proteins. (**C**) Left: superimposition of the SAM dependent methyltransferase domain (MTase) of TrmS (Alphafold model) and NscB1 (PDB 3I53). Middle: Putative tRNA-binding model of TrmS (Alphafold model). The structure of tRNA^Asp^ is represented by light-green cartoon. Right: The structure modeling of TrmS. The sticks boxed represent the conserved residues in the proximity of SAH. Electrostatic potential mapped on the surface of each protein in which positive charges are shown in blue, negative charges in red, and neutral charges in white. SAH, [4Fe-4S]^2+^ cluster, 5′-dA, and SAM are shown in stick as indicated and colored by atom type.

In addition to assigning *in vivo* substrate specificities, the epitranscriptome library screen provided functional annotation of RNA-modifying enzymes and revealed evolutionarily distinct dual-function enzymes with nonredundant activities. For example, we identified PA14_14340 as a dual specificity m^6^A methyltransferase for both rRNA and tRNA. PA14 lacks the tRNA m^6^A writer YfiC known to modify A37 of tRNA_1_^Val^ in *E. coli* [48]. Instead, PA14_14340 is responsible for 99% of m^6^A in tRNA and 45% in rRNA, with the remaining 55% of m^6^A in rRNA attributed to PA14_66340 (RlmJ) (Supplementary Fig. S10A and C). PA14_14340 shares significant sequence and structural similarity with RlmF (Supplementary Fig. S11A), an m^6^A methyltransferase specific for A1618 of 23S rRNA in *E. coli* [49] (Supplementary Fig. S10A and C). In the protein-tRNA^Val^ docking model in Fig. 4A, PA14_14340 forms a positively charged concave surface near the *S*-adenosyl-L-homocysteine (SAH)-binding site surrounded by three polypeptide loops (boxed in Fig. 4A) that likely interact with the tRNA^Val^ anticodon stem loop (ASL), as with human METTL16 [50]. PA14_14340 thus represents a dual-function RNA methyltransferase that shares m^6^A synthesis activity with RlmJ.

Similarly, we identified two enzymes responsible for m^2^A in tRNA. Loss of *rlmN* (*PA14_14830*) eliminated m^2^A in rRNA entirely and reduced it in tRNA by 80%, with *PA14_40730* responsible for the remaining 20% (Supplementary Fig. S10A and B). Here we name the enzyme encoded by *PA14_40730* as TrmV, a tRNA (adenine(37)-C(2))-methyltransferase (EC 2.1.1.192), TrmV-type. Since m^2^A levels in tRNA are ten-times higher than rRNA (Supplementary Fig. S10G), the 20% reduction in m^2^A in tRNA in *trmV* is unlikely caused by rRNA contamination. In *E. coli* and other bacteria, RlmN is the only m^2^A enzyme and modifies both rRNA and tRNA [51, 52]. Based on phylogenetic analyses (Supplementary Fig. S11B) and significant sequence and structural similarity between TrmV and RlmN (Supplementary Fig. S11C), it is possible that TrmV evolved by duplication of RlmN and a shift in substrate specificity. Structural comparison shows [1] a positively charged groove in both proteins accommodating the anticodon stem-loop of tRNA with A37 in the active site [53] (Fig. 4B and Supplementary Fig. S11C), conserved residues Met168 and Cys346 (RlmN numbering) likely forming a transient thiosulfuranyl radical required for both radical *S*-adenosyl-L-methionine (SAM) proteins (boxed, Fig. 4B), and amino acids unique to RlmN or TrmV (e.g. Arg198 in RlmN; boxed, Supplementary Fig. S11C) distinguishing tRNA and rRNA.

The PA14 epitranscriptome screen also uncovered a novel RNA methyltransferase activity that represents an evolutionary nonorthologous displacement of *E. coli* TrmH [54]. PA14_68100 catalyzed all *G*_m_ and ∼25% of *C*_m_ in tRNA, but not in rRNA (Supplementary Fig. S10A, D, and E). Here, we name the enzyme encoded by *PA14_68100* as TrmS, a tRNA (guanidine(18)-2′-O)-methyltransferase (EC 2.1.1.34), TrmS-type. RlmB (PA14_65190) catalyzed *G*_m_ in rRNA (Supplementary Fig. S10A and D). While *E. coli* TrmH is strictly a tRNA guanosine-2′-*O*-methyltransferase of the Class I SPOUT methyltransferase family [54], TrmS contains a Class IV Rossmann fold-like structure (Fig. 4C and Supplementary Fig. S12A) and has apparently evolved in *Pseudomonadota* differently from TrmH and RlmB (Supplementary Fig. S12B and C).

### The knockout library epitranscriptome dataset provides an opportunity to identify gene

Our screening findings further suggest the presence of m^6^_2_A in PA14 tRNA and implicate an enzyme specific to it. The loss of *PA14_07730* (*rsmA*), m^6^_2_A methyltransferase for 16S rRNA in *E. coli* [55], induces the absence of m^6^_2_A in rRNA, while with 8.6% remaining in tRNA (Supplementary Fig. S10A and F). Given m^6^_2_A is 10 times more abundant in rRNA than tRNA (Supplementary Fig. S10G), it was previously assumed to be specific to rRNA. However, our results indicate the existence of alternative enzyme is responsible for m^6^_2_A in PA14 small RNA, instead of RsmA.

Finally, we functionally confirmed several tRNA modifying activities in PA14. PA14_16930 shares strong sequence and structural similarity with ct^6^A-catalyzing *E. coli* cysteine desulfurase CsdA [56, 57] (Supplementary Fig. S13C and D) and its loss abolished ct^6^A (Supplementary Table S6 and Supplementary Fig. S13B). Similarly, deletion of *PA14_17650* caused a ∼70% reduction of acp^3^U. This mirrors the function of the sequence- and structurally-similar *E. coli* TapT (YfiP) as a tRNA-uridine aminocarboxypropyltransferase (Supplementary Fig. S13E and F) and reinforces recent findings in PA14 [58–60].

A single screening experiment thus allowed functional annotation of many RNA-modifying activities and led to discovery of new activities. More important, however, are revelations of pathways linking environmental changes to changes in the tRNA epitranscriptome, as discussed next.

### Epitranscriptome regulatory networks

**Figure 5.**
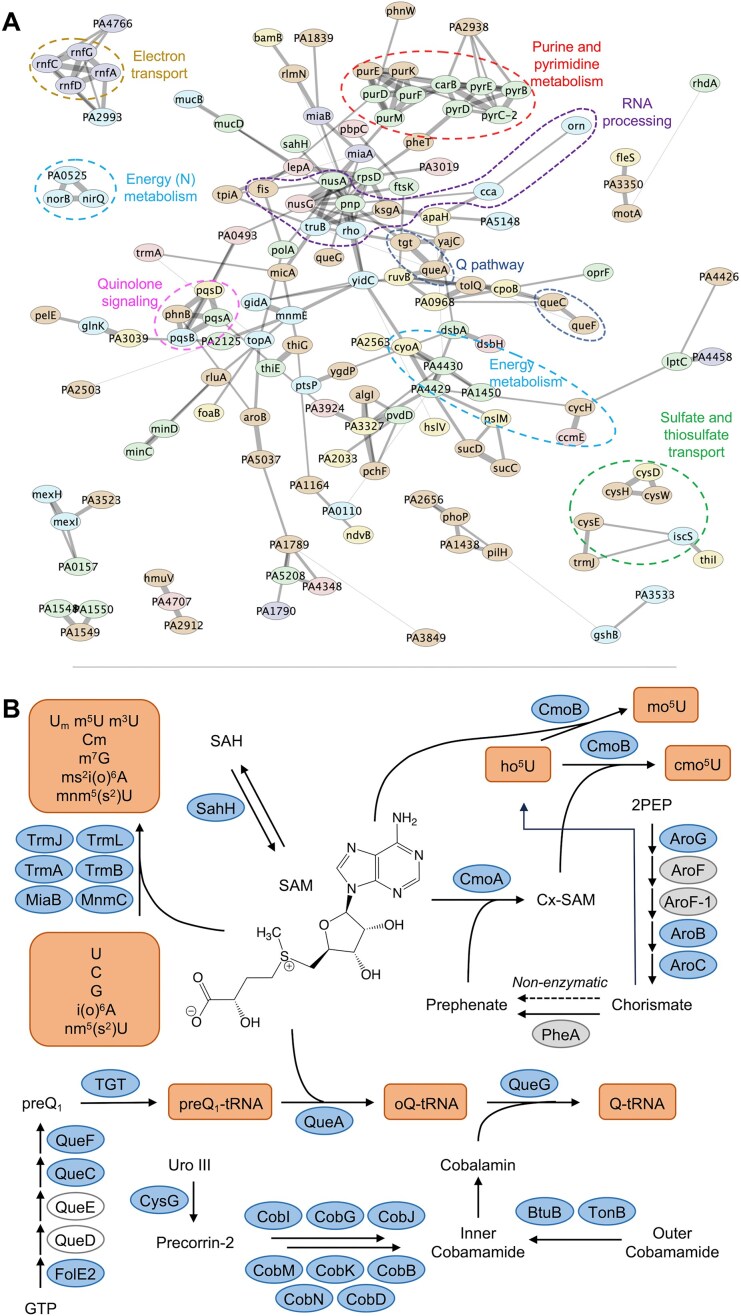
Genome-scale network analysis of tRNA modification changes in the PA14 mutant library reveals epitranscriptome engagement with metabolic, regulatory, and signaling pathways. (**A**) A protein interaction network (STRING) for 143 genes causing significant changes in the levels of 30 modifications. Proteins are depicted as nodes (circles with gene names) with colors corresponding to the mutant clusters in the heatmap in Fig. 3A. Edge thickness (lines linking nodes) correlates with the STRING protein interaction confidence score. For visualization, only clusters with ≥3 proteins and edges with confidence scores ≥0.5 are shown (143 of 321 proteins affecting modification levels); 178 genes are not shown. (**B**) Epitranscriptome analysis reveals a SAM-centric gene network influencing tRNA modification biogenesis. SAM and carboxyl-SAM (Cx-SAM) are cofactors for dozens of RNA methyltransferases. For example, CmoB differentially catalyzes mo^5^U and cmo^5^U depending upon availability of SAM and Cx-SAM, respectively. Cx-SAM levels are determined by levels of prephenate from shikimate pathway dynamics. SAM is also involved in the biogenesis of Q, together with multiple tRNA-modifying proteins and the QueG cofactor cobalamin. Blue ellipses: proteins significantly altering tRNA modifications noted in orange boxes. Grey ellipses: proteins not affecting tRNA modification levels. Clear ellipses: proteins encoded by genes absent in the PA14 library. dashed arrow linking chorismate to prephenate denotes non-enzymatic conversion. PEP: 2-phosphoenolpyruvate.

Analysis of the 313 genes causing significant changes in tRNA modification levels (Fig. 3A) revealed multiple networks indirectly regulating specific modifications and related families, as revealed in the six clusters in Fig. 3A (left) and more specifically by functional groupings in the STRING protein interaction network in Fig. 5A. This functional clustering starts proximal to the RNA-modifying enzymes, most of which have cofactors with metabolic pathways that affect RNA modification levels. For instance, loss of *sahH* (*PA14_05620*), which encodes SAH hydrolase, caused a decrease in SAM-dependent modifications such as U_m_, C_m_, m^5^U, m^3^U, m^7^G, ms^2^i^6^A, ms^2^io^6^A, mnm^5^s^2^U, cmo^5^U, mcmo^5^U, and Q, but an accumulation of preQ_1_ (Fig. 5B and Supplementary Table S6). Loss of SahH causes accumulation of SAH, a nonselective feedback inhibitor for many methyltransferases [61] and the demethylated product of methyltransferase cofactor SAM. This explains reduced levels of methylation-based modifications.

Expanding outward to metabolic pathways generating the cofactors, another example involves carboxy-*S*-adenosylmethionine (Cx-SAM), the preferred cofactor competing with SAM in CmoB-mediated synthesis of cmo^5^U and mcmo^5^U (Fig. 5B) [62]. In the absence of Cx-SAM, CmoB uses SAM to synthesize mo^5^U, albeit at a reduced rate. Cx-SAM is synthesized by CmoA from prephenate derived from the shikimate pathway for aromatic amino acid synthesis (Fig. 5B). This explains why loss of *aroB* and *aroC* in the shikimate pathway reduced levels of cmo^5^U and increased ho^5^U and mo^5^U (Figs 3A and 5B; Supplementary Fig. S14 and Supplementary Table S6). While PheA converts chorismate to prephenate, its loss did not fully eliminate cmo^5^U (Fig. 5 and Supplementary Fig. S14), likely due to slow nonenzymatic conversion [63]. Additionally, PA14 possesses at least three redundant 3-deoxy-d-arabinoheptulosonate-7-phosphate (DAHP) synthases in the shikimate pathway: AroF, AroF-1, and a putative AroG (PA14_27330) (Fig. 5B and Supplementary Table S8). This redundancy may explain why deletion of only AroG had a slight impact on mo^5^U and cmo^5^U levels (Supplementary Table S6 and Supplementary Fig. S14).

Similarly, deletion of genes involved in cobalamin translocation (*btuB, tonB*) and biogenesis (*cysG, cobB/D/G/I/J/K/M/N/H*) impeded cobalamin-dependent QueG conversion of epoxyqueuosine (oQ) to Q (Fig. 5) and caused oQ accumulation (Supplementary Table S6). Interestingly, loss of the anaerobic cobalamin biogenesis enzyme CysG led to the accumulation of oQ under aerobic conditions, suggesting that the CobA aerobic counterpart might not fully compensate for loss of CysG [64].

We next used the STRING database [22] to evaluate interactions among the 143 modification-altering proteins (nodes/circles with gene names in Fig. 5A). In several instances, the six clusters of mutant strains in Fig. 3A translate to groups of functionally related proteins in Fig. 5A (node colors = heat map clusters). This functional relatedness is emphasized when Gene Ontology (GO) categories are overlaid on the network (dashed circles in Fig. 5A), which suggests physiological regulatory roles for tRNA modifications. Regulatory potential is also suggested by quantifying the number of gene mutations that affect a modification level, as shown in Supplementary Fig. S15, which adds gene function to the gene-modification links in Fig. 3A. For example, i^6^A was altered by 62 genes (nodes) and connected with io^6^A through another 37 genes, more than any other pair of modifications. The pairs ct^6^A-t^6^A and ms^2^i^6^A-ms^2^io^6^A were connected by 17 and 11 nodes, respectively, consistent with their shared synthesis pathways. As a single gene, *PA14_05620* (*sahH*) connects to the most modifications [11], consistent with its role in recycling the SAH product of the RNA methyltransferase SAM cofactor (Fig. 5B).

The regulatory potential for the tRNA epitranscriptome is also apparent in connections between individual modifications. For example, 34 of 41 nodes connected to ms^2^i^6^A (Supplementary Fig. S15) are also connected to other modifications, including i^6^A, m^6^t^6^A, ms^2^io^6^A, m^5^C, m^3^U, and m^2^G. This suggests a role for ms^2^i^6^A as a precursor in tRNA maturation or as part of a signaling network linking metabolic shifts to translation, for example. This latter point is illustrated next with the i^6^A family of tRNA modifications.

### The MiaB Fe-S cluster integrates metabolic signaling

**Figure 6.**
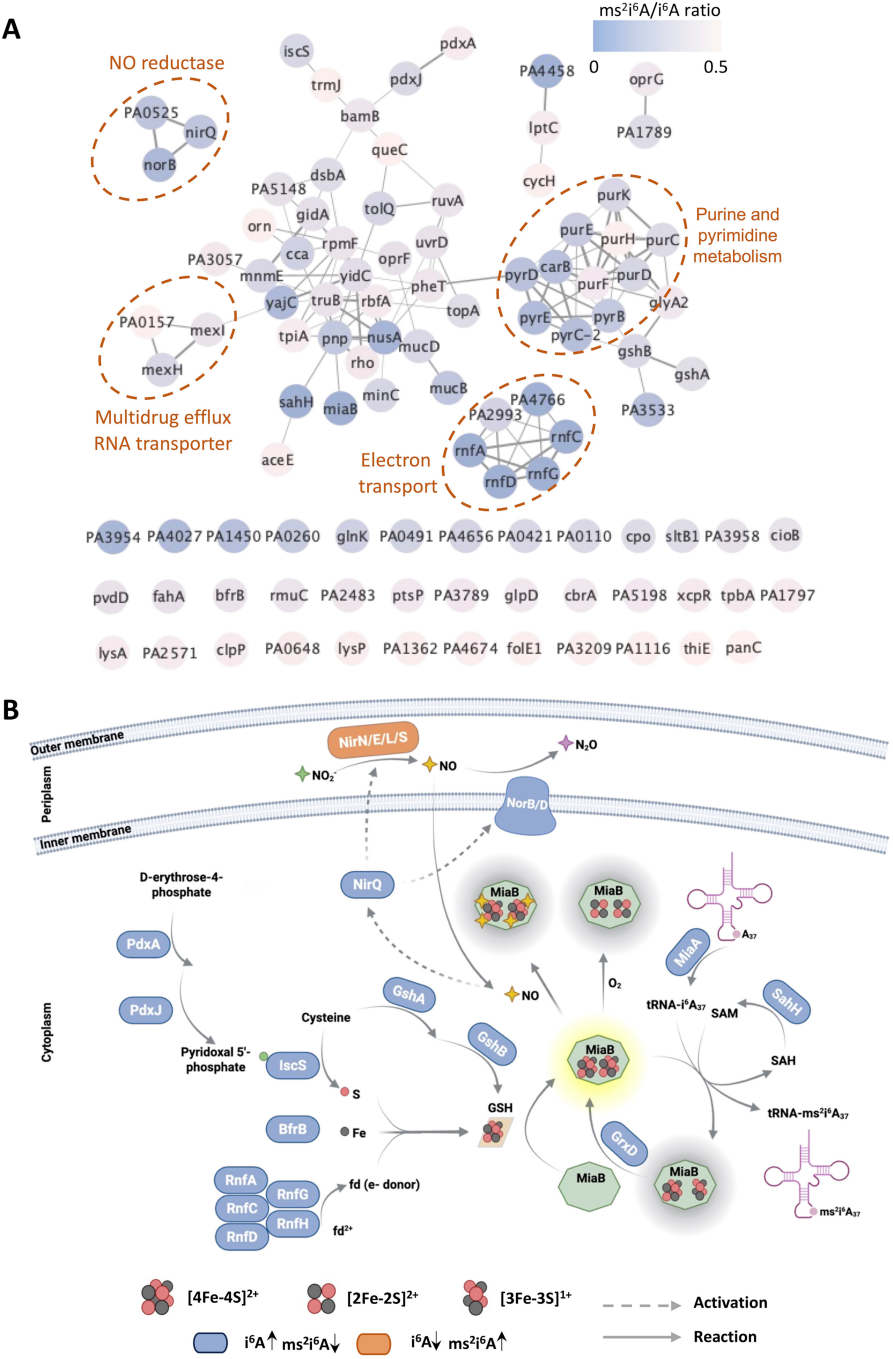
Visualization of gene networks influencing MiaB activity. (**A**) Here, we used the ratio of ms^2^i^6^A to i^6^A (ms^2^i^6^A/i^6^A < 0.5) as metric to create an interaction network of proteins affecting MiaB activity. The genes are annotated according to *Pseudomonas aeruginosa* PAO1 strain. Node color indicates the ms^2^i^6^A/i^6^A ratio (key upper right), the edge width (connecting lines) correlates with the STRING confidence score for the protein interactions; confidence scores ≥0.4 are displayed. Functional categories are encircled with a dashed line. (**B**) Diagram illustrating the metabolic and regulatory pathways centered on Fe-S clusters affecting MiaB activity and ms^2^i^6^A biosynthesis, as inferred from Fig. 5A. Proteins highlighted in blue represent gene knockouts that lead to MiaB dysfunction, increasing i^6^A levels and decreasing ms^2^i^6^A. Conversely, proteins in orange represent gene knockouts that lower NO levels, thereby enhancing MiaB activity to cause decreased i^6^A and increased ms^2^i^6^A levels. The image was created with Biorender.com and available at https://biorender.com/4rhsiff.

Levels of i^6^A-related modifications (i^6^A, io^6^A, ms^2^i^6^A, and ms^2^io^6^A) are affected by many genes in PA14 (Fig. 3A and Supplementary Fig. S15), suggesting that their catalytic enzymes (MiaABE, Fig. 3B) function as regulatory nodes in signaling pathways linked to translational regulation of gene expression. We explored this with MiaB, which inserts sulfur in i^6^A to form ms^2^i^6^A [45] and, as shown here, converts io^6^A to ms^2^io^6^A (Supplementary Tables S6 and S7). Here, we analyzed PA14 genes that caused the ms^2^i^6^A/i^6^A ratio to fall below 0.5, indicating inhibition of MiaB activity. A map of protein-protein interactions for these genes (Fig. 6A) reveals a complicated but significant (PPI enrichment *P*< 1.0e^−16^) network of 104 gene nodes with 132 edges, reflecting cellular processes such as iron-sulfur (Fe-S) cluster assembly and maintenance, nitric oxide (NO) detoxification, and oxidative stress response. It is immediately apparent that the MiaB Fe-S cluster plays a key role in this network, as indicated by its strong association with Fe-S cluster biogenesis and maintenance proteins (Fig. 6B): Cysteine desulfurase IscS and PdxA/J enzymes synthesizing IscS cofactor pyridoxal 5′-phosphate (PLP); BfrB for iron storage; the RnfA/C/D/G/H family as the ferredoxin/electron donor to Fe-S clusters; GshA/B for Fe-S cluster export; and GrxD for maintenance and repair of Fe-S cluster proteins. Loss of these proteins inhibited conversion of i^6^A to ms^2^i^6^A, especially for proteins in the Rnf complex that caused the complete disappearance of ms^2^i^6^A.

The signaling network expands to gene clusters in Fig. 6A. Intrinsic NO metabolism and NO exposure (e.g. activated macrophages) regulate many pathogenesis pathways in the facultative anaerobe *P. aeruginosa* at low oxygen levels by activating the dissimilative nitrate respiration regulator (DNR) transcription factor [65]. Among genes involved in NO metabolism (*nirENLQS*, *norBD*), loss of *nirQ*/*norB*/*norD* leads to accumulation of NO that is well established to disrupt Fe-S clusters [66]. Conversely, loss of nitrite reductase genes *nirN*, *nirE*, *nirL*, and *nirS* reduced i^6^A and io^6^A levels and slightly increased ms^2^i^6^A (Supplementary Table S6), consistent with increased MiaB activity. Finally, reactive oxygen species (ROS) oxidize and disrupt Fe-S clusters [67], so it is not surprising that two gene clusters were associated with elevated i^6^A and io^6^A and decreased ms^2^i^6^A and ms^2^io^6^A: ROS response proteins and proteins the loss of which increases ROS. The former are illustrated in Fig. 6B by glutathione biosynthesis enzyme GshA/B, redox enzyme glutaredoxin GrxD, vitamin B6 synthesis enzymes PdxA/J, alkanesulfonate monooxygenase SsuD (PA14_12710), pyrimidine biosynthesis (PyrC/D/E, PA14_05250/24640/70370), sigma factor *algU* regulator MucB (PA14_54410), and transcriptional regulator OxyR (PA14_06400). In parallel, ROS increase with loss of proteins for transcription termination factors NusA (PA14_62770) and Rho (PA14_69190), multidrug efflux protein MexH/I (PA14_09520, PA14_09530), cmnm^5^s^2^U writer MnmE/G (PA14_73400, PA14_73370), and ribonuclease Orn (PA14_65410) (Fig. 6A).

## Discussion

Application of “omic” technologies has revealed the systems-level function of well-known tRNA modifications in translational regulation of gene expression [5, 7, 68–70] and adaptation to environmental changes and stress [5, 68, 69, 71]. However, the gene and signaling networks regulating levels of tRNAs and their modifications have largely eluded definition, in part due to the lack of technology for large-scale functional genomics studies. To identify epitranscriptome regulatory networks, we developed a HT tRNA modification analytical platform and applied it the 5850-strain PA14 transposon insertion library. The results provide the first comprehensive analysis of tRNA modification regulatory networks and open the door to sequencing- and LC-MS-based ribonucleomics and epitranscriptome mapping in cells and tissues.

Driven by RNA instability [72], large sample number, and small RNA quantities, the platform required optimization of all steps—cell lysis, RNA purification, RNA processing, MS analysis, and MS data processing—to achieve high quantitative precision (CV < 25%) and accuracy. During adaption of this approach for RNA purification, we identified several critical parameters, including the importance of (i) GITC for cell lysis, (ii) magnetic beads composition, buffer components, and isopropanol elution for size-based RNA resolution, and (iii) liquid handler workflows and plasticware for reproducible RNA quality. This workflow, which avoids volatile solvents such as phenol and chloroform, eliminates the need for fume hood handling and significantly reduces hazardous waste, offering a safer and more practical approach for routine laboratory use. The choice of carboxylate-coated magnetic beads, widely used for DNA purification by molecular crowding size selection [73], was based on broad compatibility with cells and tissues. Care must be taken in interpreting results from size-based RNA purification given modifications present in co-purifying fragments of rRNA, mRNA, and long non-coding RNAs (lncRNAs) (e.g. m^5^C, m^6^A, Y, G_m_, C_m_, U_m_, and A_m_). Our focus on tRNA partly reduces such interference due to the much higher density of modifications in tRNA compared to other RNAs [15], while m^4^C and m^6^_2_A appear to be limited to rRNA and serve as an rRNA degradation metric [74]. However, as illustrated for Rlm family methyltransferases (Supplementary Fig. S11), the platform can be readily applied to rRNA and other large RNAs to facilitate functional annotation of multi-substrate RNA-modifying enzymes. Care must also be taken to avoid RNA processing artefacts, such as use of deaminase inhibitors [75] to prevent deamination of C (uridine), G (xanthosine), and A (inosine, Supplementary Fig. S6A), antioxidants to prevent loss of oxidation-sensitive modifications (e.g. ho^5^U, ho^5^C, 76)), and avoidance of centrifugal filtration devices that can bias toward nonpolar RNA modifications (Supplementary Fig. S6B) [77]. Overall, the optimized UPLC-MS/MS method minimized analysis time and maximized ribonucleoside resolution in 6-min runs for 40 modifications, which amounted to ∼900 h for the 5850-strain library. The platform can be adapted to modifications in all forms of RNA in any organism or tissue, with future enhancements of increased MS sensitivity for smaller sample input, shortened LC-MS/MS turnaround time for screening larger cell or chemical libraries, and AI-enabled tools for efficient peak detection in large MS datasets.

Application of the epitranscriptome platform allowed annotation of gene function but more importantly revealed tRNA modification gene networks with significant regulatory potential as stress sensors and response effectors. Anticodon loop modifications at the wobble position (Q, cmo^5^U, mcmo^5^U, and ho^5^U) and position 37 (i^6^A, io^6^A, ms^2^i^6^A, and ms^2^io^6^A) stood out prominently in this regard (Figs 3A and 5A), consistent with their roles in codon recognition linked to stress-induced tRNA reprogramming and codon-biased translation of stress response mRNAs [5, 71, 78]. The xo^5^U wobble modification family illustrates this point. Ruling out a proposed role for chorismate in ho^5^U synthesis [79], we observed that loss of synthetic genes AroBCG for the prephenate precursor chorismate increased ho^5^U and mo^5^U levels and reduced cmo^5^U and mcmo^5^U (Fig. 5 and Supplementary Fig. S14). It is thus possible that cellular decreases in prephenate, due to increased demand for aromatic amino acids or secondary metabolites from the shikimate pathway, will cause reduced levels of Cx-SAM and thus reduced levels of cmo^5^U and mcmo^5^U modifications in tRNAs with UNN anticodons (Ala-UGC, Ser-UGA, Pro-UGG, and Thr-UGU for mcmo^5^U; Leu-UAG, Val-UAC for cmo^5^U; 80). Since cmo^5^U and mcmo^5^U expand the codon repertoires of these tRNAs to include G-ending codons [81, 82], we would predict that reduced levels of these modifications would shift translation to favor mRNAs enriched with A- and T-ending codons.

The dynamics of MiaB-mediated i^6^A modifications further illustrate the regulatory potential of tRNA-modifying enzymes. Just as there are Fe-S cluster-containing transcriptional regulators that sense O_2_ and iron [83], we propose Fe-S cluster enzymes as translational regulators given their roles in forming RNA modifications [84]. MiaB illustrates this point with potential involvement in sensing changes in Fe-S biogenesis and repair, nitric oxide metabolism, and redox regulation. Other Fe-S cluster RNA-modifying proteins, such as QueG (Q), TtcA (s^2^C), and RlmN (m^2^A), were not affected by loss of genes that altered MiaB activity. For example, deletion of *nirQ, norB*, or *norD* did not alter Q, s^2^C and m^2^A levels but did affect i^6^A-related modifications (Supplementary Table S6). One potential reason is that MiaB is unusual in containing two [4Fe-4S] clusters, one mediating formation of the adenosine radical intermediate and the other forming an unstable [3Fe-4S] intermediate for methylthio group transfer [85, 86]. The resulting [3Fe-3S] must be repaired to restore MiaB activity. The Fe-S clusters of QueG, TtcA, and RlmN, on the other hand, remain intact during transfer of an electron, sulfur, or methyl group, respectively [87–89]. With i^6^A-based modifications located in tRNAs that read UNN codons [90], MiaB dependence on Fe-S biogenesis and repair, nitric oxide metabolism, and redox regulation suggests that i^6^A and ms^2^i^6^A dynamics could reprogram the tRNA pool to regulate translation of UNN-enriched stress response mRNAs, as proposed for the xo^5^U modifications and observed in diverse organisms [6, 7]. Indeed, fluctuations in ms²i^6^A levels have been linked to altered translational fidelity and adaptive mutation during stress conditions [91], while MiaB has been shown to regulate networks such as the Type III secretion system in *P. aeruginosa* [92] and MiaA-mediated stress-responsive proteins in *E. coli* [93]. The central role of MiaB in regulating i^6^A family modifications as an integrator of diverse metabolic changes raises the question of why *miaB* is not an essential gene. Among many possible explanations, partial decoding fidelity by i^6^A might buffer the impact of losing ms^2^i^6^A and activation of alternative stress-response pathways may compensate for MiaB loss. The results from this PA14 library screen open the door to a host of testable hypotheses about the regulatory functions of tRNA modifications.

In summary, we developed a robust and HT approach to quantitative analysis of the tRNA and rRNA epitranscriptomes. The platform revealed layers of gene–gene interactions that position tRNA-modifying enzymes and their cofactors as nodes in signaling networks. Focused multi-omic analyses with modification-specific subsets of PA14 knockouts will further elaborate the signalling networks and reveal compensating regulatory mechanisms. The platform can also be readily adapted to large collections of human cells and tissues, such as the hundreds of cancer cell lines in the DepMap [12], as a platform for drug discovery by whole-cell phenotypic or target-based screening of compound libraries, and for biomarker discovery. Finally, adapting the platform to other RNA analytical methods, such as small RNA processing for NGS library preparation for sequencing-based modification maps and quantifying small RNAs [94], will advance our understanding of the RNome.

## Supplementary Material

gkaf696_Supplemental_Files

## Data Availability

The mass spectrometry data have been deposited to the ProteomeXchange Consortium via the PRIDE partner repository with the dataset identifier PXD053297 (http://www.ebi.ac.uk/pride). Sequencing data have been deposited in the NCBI SRA database with BioProject ID PRJNA1126677. All data analysis and data visualization R scripts are available in the FigShare repository at https://doi.org/10.6084/m9.figshare.28582445.v1.

## Supplementary data

Supplementary data is available at NAR online.
