# Supplementary material for: tRNA modification profiling reveals epitranscriptome regulatory networks in Pseudomonas aeruginosa
Source: Nucleic Acids Res. 2025 Jul 28;53(14):gkaf696. doi: 10.1093/nar/gkaf696 (PMC12300143; doi:10.1093/nar/gkaf696)
Supplement: gkaf696_Supplemental_Files [file gkaf696_supplemental_files.zip › Sun et al Supplementary Information Revised 2025 6-3.pdf]

Supplementary Information for

High-throughput tRNA modification profiling reveals epitranscriptome regulatory networks in *Pseudomonas aeruginosa*

Jingjing Sun, Junzhou Wu, Yifeng Yuan, Leon Fan, Wei Lin Patrina Chua, Yan Han Sharon Ling, Seetharamsing Balamkundu, Dwijapriya, Hazel Chay Suen Suen, Thomas J. Begley, Valérie de Crécy-Lagard, Agnieszka Dziergowska, Peter C. Dedon

Contents

• **Supplementary Fig. 1.** Workflow for LC-MS/MS data processing.

• **Supplementary Fig. 2.** Tecan EVO150 optimization

• **Supplementary Fig. 3.** Optimization and validation of the RNA purification platform applied to cultured bacteria, human embryotic kidney cells, and mouse brain.

• **Supplementary Fig. 4.** Optimizing HPLC performance.

• **Supplementary Fig. 5.** LC-MS/MS performance characteristics.

• **Supplementary Fig. 6.** Optimizing RNA sample processing conditions.

• **Supplementary Fig. 7.** Reproducibility of RNA purity for small RNA samples purified using the Tecan platform.

• **Supplementary Fig. 8.** High-resolution mass spectrometry confirmation of signals (m^6t^6A and ms^2io^6A) found by neutral loss scan in PA14 WT strain.

• **Supplementary Fig. 9.** Resolution of transposon mutagenesis artifacts in PA14.

• **Supplementary Fig. 10.** Functional annotation of genes for RNA-modifying enzymes.

• **Supplementary Fig. 11.** Comparison of PA14_14340 and PA14_40730 with RlmF-like like and RlmN-like proteins from other bacteria.

• **Supplementary Fig. 12.** PA14_68100 is a non-orthologous displacement of *E. coli* TrmH.

• **Supplementary Fig. 13.** Annotation of PA14_16930 as CsdA involved in ct^6A formation (A-D) and alignment of PA14_17650 and $TapT_{Ec}$ (E,F).

• **Supplementary Fig. 14.** Levels of ho^5U (A), cmo^5U (B) and mo^5U (C) in mutants involved in shikimate pathway.

• **Supplementary Fig. 15.** Protein-modification network of 30 RNA modifications (center square in each cluster) linked to 312 PA14 mutant genes (nodes/circles) that affect modification levels.

• **Supplementary Table 1:** Configuration of Tecan EVO150 parameters used for magnetic beads-based tRNA isolation from crude lysates.

• **Supplementary Table 2:** Dynamic MRM table for LC-MS/MS analysis of PA14 tRNA modifications.

• **Supplementary Table 3.** Protein IDs used in this study. *Separate spreadsheet.*

• **Supplementary Table 4:** Detect of limit (LOD) and Quantification of limit (LOQ) of modified ribonucleosides using the rapid UHPLC/MS method developed in this study.

• **Supplementary Table 5:** Modification table. *Separate spreadsheet.*

• **Supplementary Table 6:** PA14 library screening results. *Separate spreadsheet.*

• **Supplementary Table 7:** Verification of known tRNA modifying enzymes. *Separate spreadsheet.*

• **Supplementary Table 8:** Gene pathway list Fe-S Denitrif SAM. *Separate spreadsheet.*

• **Supplementary Results**

• **Supplementary References**

Supplementary Figures

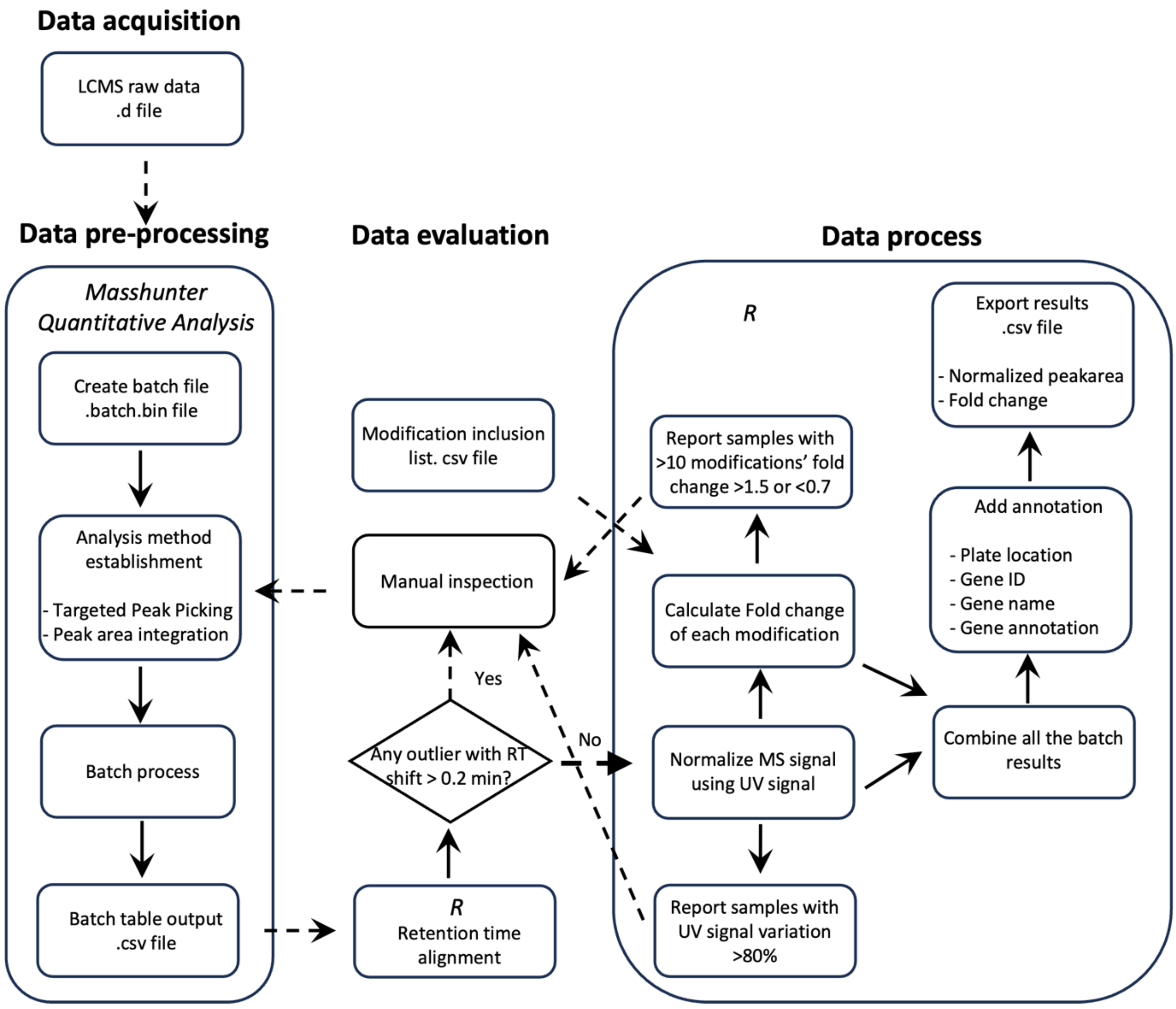

Supplementary Figure 1. Workflow for LC-MS/MS data processing. The workflow consists of three main sections: (1) Batch processing of raw UV and MS data using Agilent Masshunter software (Agilent, USA). This step involves target peak integration, identification. (2) Data evaluation and curation using R scripts. (3) Normalization of MS data based on the sum of the UV signals of the four canonical ribonucleosides, followed by statistical analysis.

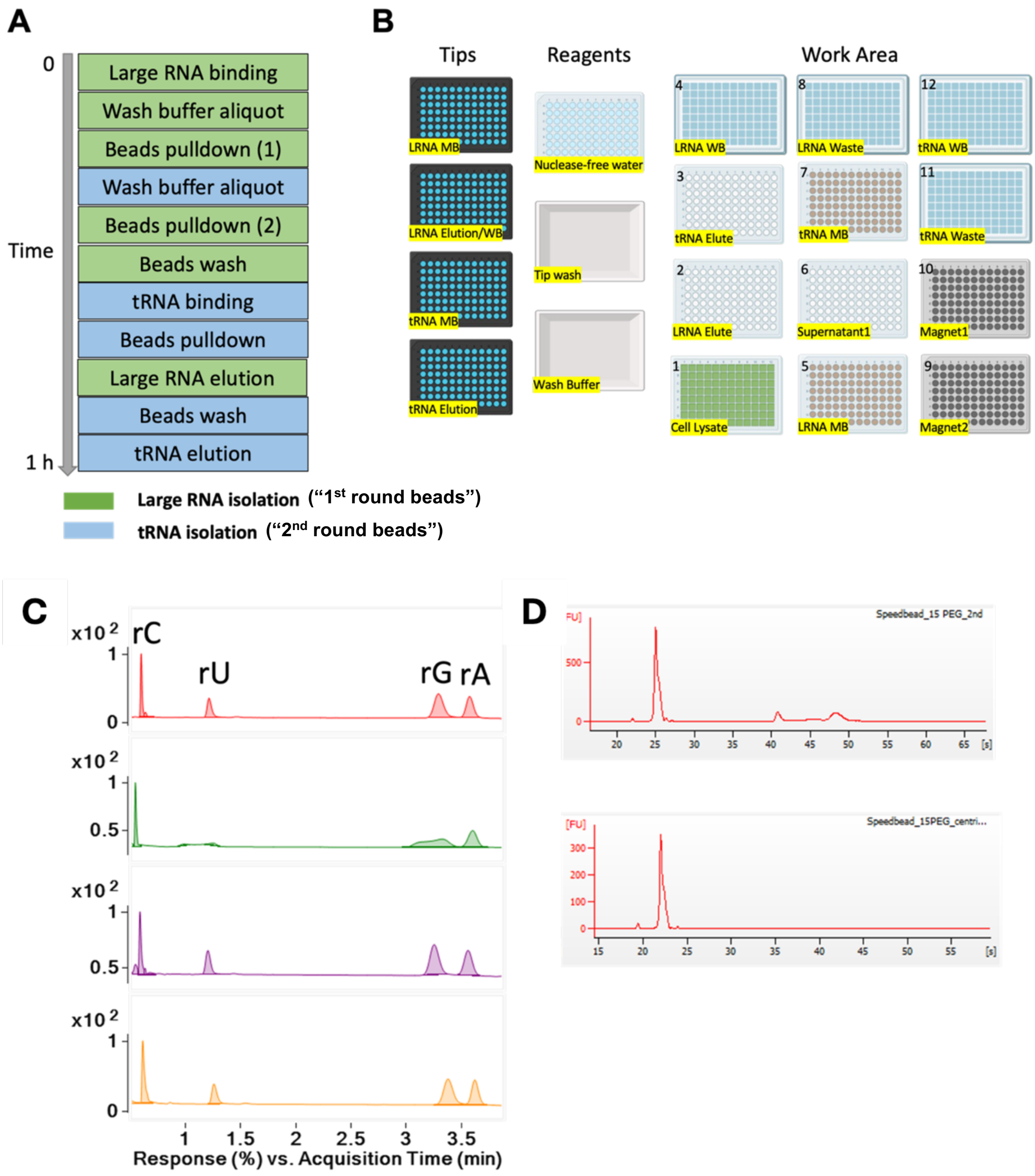

Supplementary Figure 2. Tecan EVO150 optimization. (A,B) Scheme of magnetic beads-based RNA purification workflow and the deck layout of Tecan EVO150. **(A)** EVO150 workflow for magnetic beads-based RNA purification of both large and small (<200 nt) RNA fractions. Tasks are performed in top-down order in a one-hour window. **(B)** EVO150 deck layout. The labelled icons represent different types of labware used for the RNA purification process, with deck position indicated by the number on the left top corner of work area plates. **(C)** EVO150 command optimization to minimize HPLC peak shape deterioration caused by incomplete removal of wash buffer from the final beads elution step. UV chromatograms from top to bottom: a standard consisting of hydrolyzed small RNA purified manually; small RNA purified using the unoptimized automated EVO 150 platform; previous sample dried under vacuum, redissolved, and resolved by HPLC; and hydrolyzed small RNA after command optimization for complete beads elution buffer removal. **(D)** EVO150 command optimization to eliminate rRNA contamination arising from carryover from the 1st round of beads-based purification. Pico chip bioanalyzer traces: top, purified small RNA derived from 1st round beads elution with two magnetic pulldowns; bottom: purified small RNA derived from 1st round beads elution with two magnetic pulldowns with a plate centrifugation in between the pulldowns.

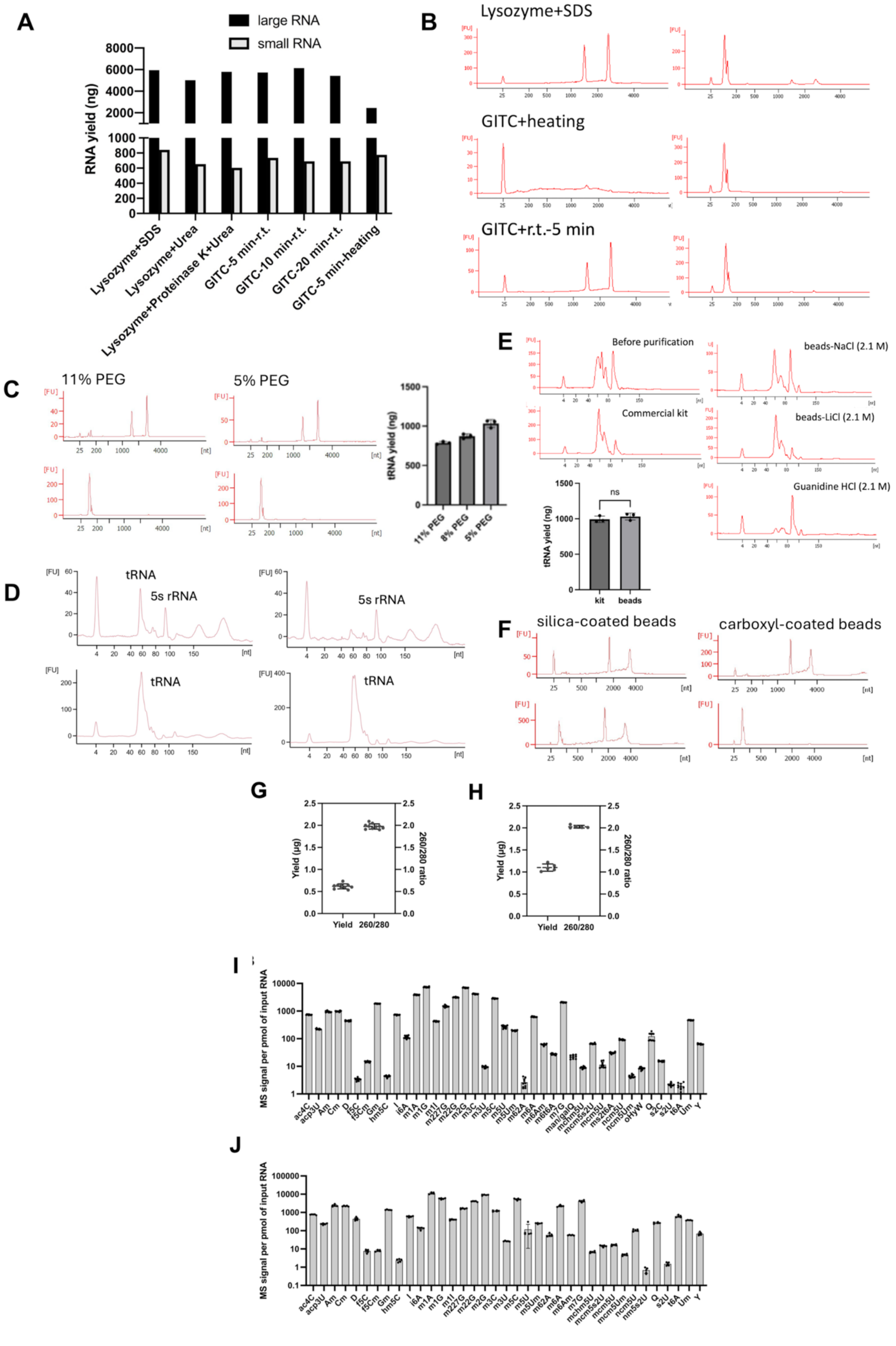
A
large RNA
small RNA
RNA yield (ng)
Lysozyme+SDS
Lysozyme+Urea
Lysozyme+Proteinase K+Urea
GITC-5 min-r.t.
GITC-10 min-r.t.
GITC-20 min-r.t.
GITC-5 min-heating
B
Lysozyme+SDS
GITC+heating
GITC+r.t.-5 min
C
11% PEG
5% PEG
tRNA yield (ng)
11% PEG
8% PEG
5% PEG
D
tRNA
5s rRNA
5s rRNA
tRNA
tRNA
E
Before purification
beads-NaCl (2.1 M)
Commercial kit
beads-LiCl (2.1 M)
Guanidine HCl (2.1 M)
ns
tRNA yield (ng)
kit
beads
F
silica-coated beads
carboxyl-coated beads
G
Yield (µg)
260/280 ratio
Yield
260/280
H
Yield (µg)
260/280 ratio
Yield
260/280
I
MS signal per pmol of input RNA
J
MS signal per pmol of input RNA

Supplementary Figure 3. Optimization and validation of the RNA purification platform applied to cultured bacteria, human embryotic kidney cells, and mouse brain. (A,B) GITC cell lysis protocol validation for PA14. **(A)** Assessment of RNA yield from 0.3 OD_{600} cells using different cell lysis protocols. Large RNA and small RNA were separated as described in Methods. *Left to right*: Lysozyme (10 mg/mL), SDS (5%) in TE buffer, pH 8.0, ambient temperature, incubate 5 min (commercial RNA extraction kit protocol for bacteria); lysozyme (10 mg/mL), urea (2 M) in TE buffer, pH 8.0, ambient temperature, incubate 5 min; lysozyme (10 mg/mL), proteinase K (0.1 mg/mL), urea (2 M) in TE buffer, pH 8.0, ambient temperature, incubate 5 min; GITC (4 M) in Tris buffer, pH 8.0, ambient temperature, vertexing 5 min/10 min/20 min (1500 rpm); GITC (4 M) in Tris buffer, pH 8.0, 65 °C, incubate 5 min. **(B)** RNA integrity assessment on Agilent bioanalyzer pico chip (25-4000 nt). (C-E) PEG-8000 and salt effects on RNA resolution. **(C)** Bioanalyzer pico chip traces of isolated RNA species in presence of 11% PEG-8000 (*Left upper*: 1st round beads eluents; *Left lower*: 2nd round eluents) and 5% PEG-8000 (*Middle upper*: 1st round beads eluents; *Middle lower*: 2nd round eluents). *Right*: tRNA yield with 11% vs. 5% PEG-8000. **(D)** Bioanalyzer small chip traces of large and small RNAs purified in the absence (*Left upper*: eluents from 1st round beads; *Left lower*: 2nd round eluents) and presence of GITC (*Right upper*: 1st round beads eluents; *Right lower*: 2nd round eluents). Conclusion: GITC improves resolution of 5s rRNA and tRNA. **(E)** Bioanalyzer small chip traces of total RNA before purification (*left upper*), small RNA species purified by commercial kit (*left lower*), beads in 2.1 M NaCl (*right upper*), 2.1 M LiCl (*right middle*) and 2.1 M guanidine HCl (*right lower*). Under acidic conditions (pH 5.3), carboxyl-magnetic beads did not bind RNA, while basic conditions (pH 8.0) caused RNA hydrolysis. A pH of 7.5 was chosen for these studies. The tRNA yield from the beads-based method is comparable to a commercial kit (Student's t-test, n=3). **(F)** Silica-coated vs. carboxyl-coated beads for RNA isolation from mouse brain samples. Bioanalyzer pico chip traces of 1st (*upper*) and 2nd (*lower*) round silica beads eluents (*left*) and carboxyl beads eluents (*right*). Application of the platform to **(G, I)** GripTite 293 MSR human embryotic kidney cells (HEK293) and **(H, J)** samples of mouse C57BL/6 brain tissue (9-month-old, n=5 mice). **(G, H)** RNA yield and purity from **(G)** HEK293 and **(H)** mouse brain. **(I, J)** The level of each modification is presented as log_{10} of the MS signal per pmol of input hydrolyzed RNA. Data are plotted with 2 biological replicates (n=10).

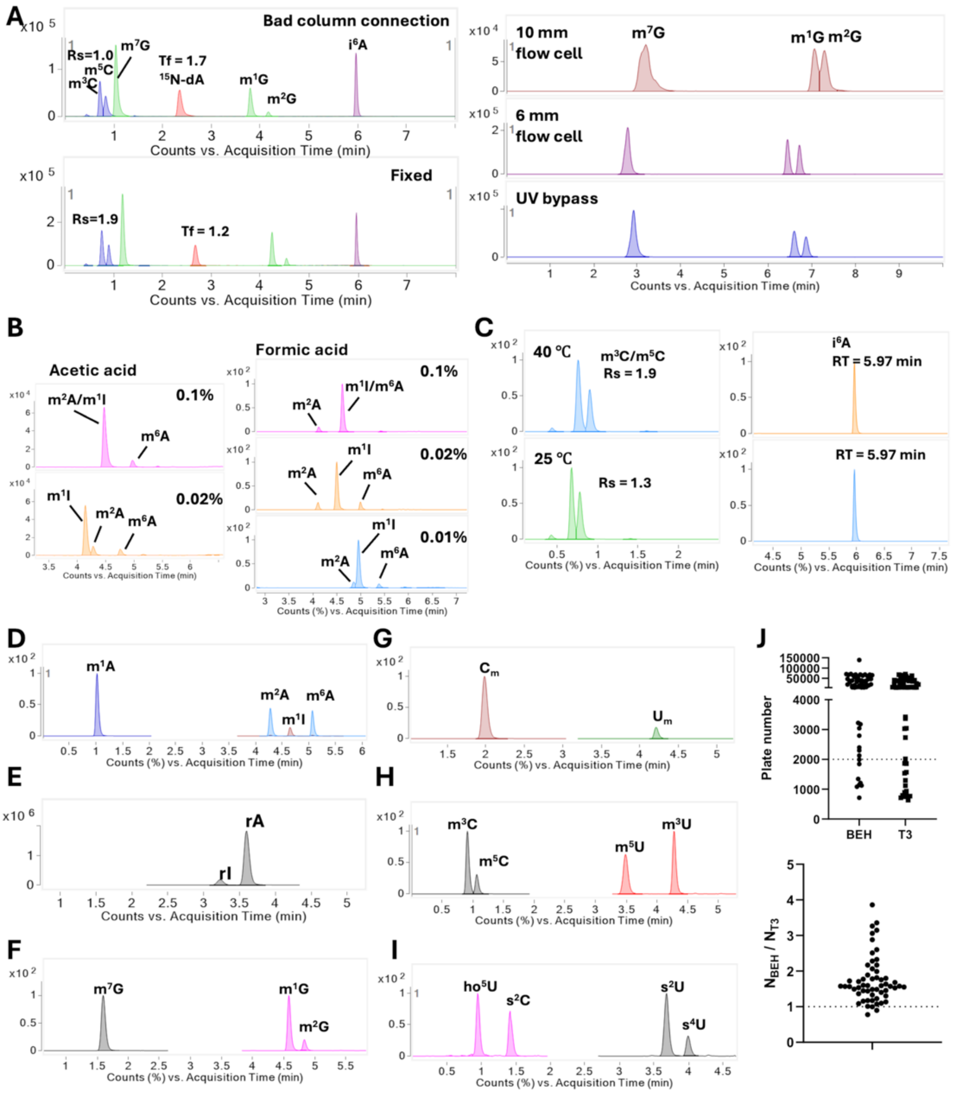

Supplementary Figure 4. Optimizing HPLC performance. (A) Minimizing HPLC column dead volume improves peak resolution. *Left*: The upper panel shows that dead volume due to longer tubing and a larger connection causes peak tailing and reduces resolution. This is solved in the lower panel. *Right:* Dead volume in a standard 10 mm flow cell (13 µL; upper) reduces peak resolution for 3 isobaric m^xGs. This was solved with a lower volume bypass UV detector (middle) or high pressure 6 mm flow cell (1.7 µL). **(B)** Optimizing mobile phase pH to resolve m^1I, m^2A, and m^6A due to similar MRM transitions (m^1I *m/z* 283>151, m^2A/m^6A *m/z* 282>150). *Acetic acid:* top, 0.1%; bottom, 0.02%. *Formic acid (FA):* top, 0. 1%; middle, 0.02%; bottom, 0.01%. 0.02% formic acid proved optimal. **(C)** Optimizing column temperature for resolution of m^3C/m^5C (left) and i^6A (right). *Upper*: 25 ºC. *Lower*, 40 °C. 25 °C offers separation of isobaric m^3C and m^5C without slowing elution. **(D-I)** HPLC resolution of ribonucleosides with the same or similar CID transitions. **(D)** m^1I, m^2A, m^6A: *m/z* 282>150, m^1I *m/z* 283>151. **(E)** rI, *m/z* 269>137; rA, *m/z* 268>136. **(F)** m^7G, m^1G and m^2G: *m/z* 298>166. **(G)** Cm, *m/z* 258>112; Um, *m/z* 259>113; **(H)** m^3C, m^5C: *m/z* 258>126; m^3U, m^5U: *m/z* 259>127. **(I)** ho^5U, *m/z* 261>129; s^2C, *m/z* 260>128; s^2U, s^4U: *m/z* 261>129. **(J)** The Waters BEH C18 column (50 mm, 2.1 mm, 1.7 µm) was chosen over the Waters HSS T3 (100 mm, 1 mm, 1.8 µm) column for shorter run times and better resolution, reflected in the larger number of theoretical plates (plate number, N). BEH conditions: 0.35 mL/ min, 330 bar, 25 °C, 100% solution A (water, 0.02% FA) for 2 min, followed by 2-4 min at 0%-8% solution B (70% acetonitrile, 0.02% FA), and from 4-5.9 min at 8%-100% B. TSS conditions: 0.25 mL/ min, 650 bar, 40 °C, 0-1.5 min, 0-3% solution B (70% acetonitrile, 0.02% FA); 1.5-5 min, 3-14% B; 5-5.10, 14-100% B; 5.1-8 min, 100 % B.

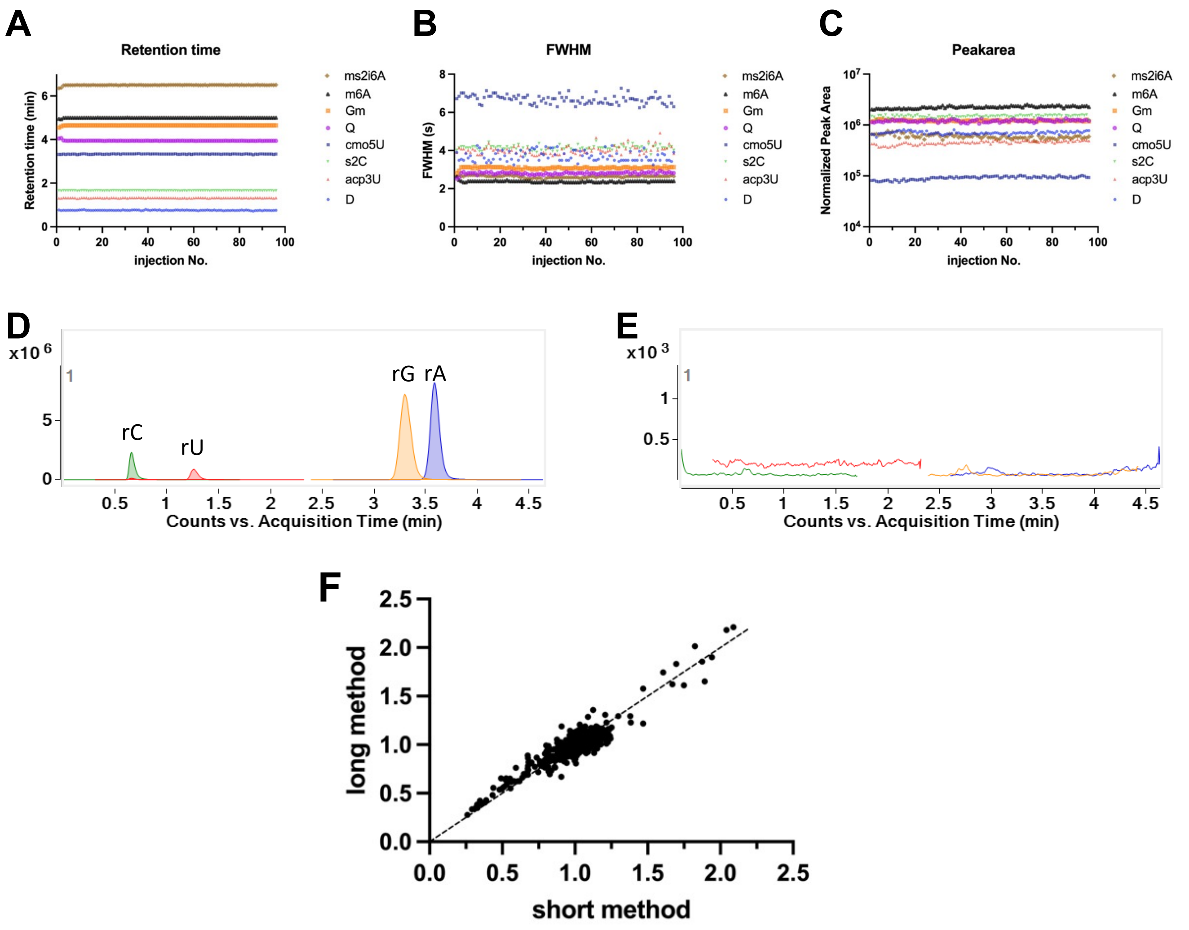

Supplementary Figure 5. LC-MS/MS performance characteristics. (A-E) LC-MS/MS method intra-day performance using the signals of RNA modifications from an injection of 200 ng hydrolysed PA14 small RNA (n = 96). A subset of 8 modifications are shown as representative in this figure due to their HPLC retention times spanning the entire analyte elution window. The statistical data in Results are calculated based on all modifications detected in the PA14 WT strain. The performance of the method during 96 injections is assessed by (A) retention time stability, **(B)** full width at half maximum peak height (FWHM), and **(C)** normalized peak area. **(D,E)** Assessment of MS signal carryover between sample injections. **(D)** Representative sample injection and **(E)** subsequent water blank injection following the sample. There is little detectable carryover. **(F)** Comparison of short and long LC-MS/MS methods. The plot shows fold-change values for 30 modifications detected in human embryonic kidney (HEK293T) cell samples (n = 16). Fold-change was calculated relative to the mean of 16 samples. The HPLC conditions for the long method (1): Phenomenex Synergi Fusion-RP C18 column (100 × 2.0 mm, 2.5 µm) coupled to an Agilent 1290 HPLC system at 35 ºC and a flow rate of 0.35 mL/min, with a gradient starting with 100% solution A (5 mM ammonium acetate, pH 5.3), followed by 0-10% solution B (acetonitrile) 0-10 min; 10%-40% solution B, 10-14 min; 40%-80% solution B, 14-15 min; 80%-90% solution B, 15-15.1 min; 90% solution B, 15.1-18 min.

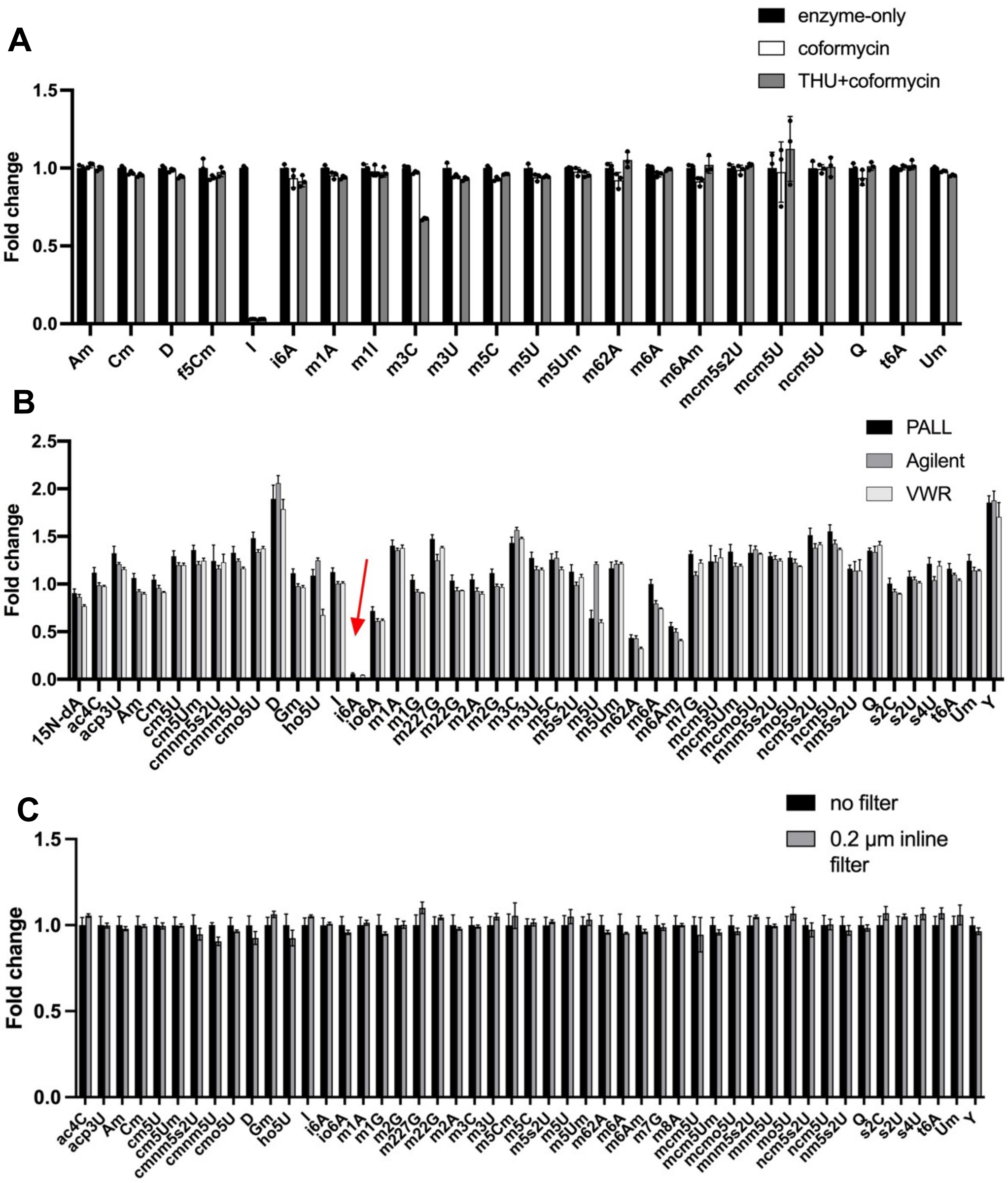

Supplementary Figure 6. Optimizing RNA sample processing conditions. (A) The effect of additives in tRNA hydrolysis enzyme cocktails on RNA modification analysis. tRNA extracted from HEK 293 cells (5 µg) was hydrolyzed in 3 different enzyme master mixes: *Enzyme-only*: 5 mM Tris, 2.5 mM $MgCl_2$, 2.5 U benzonase, 5 U CIAP, and 0.15 U PDE I; *Coformycin*: 5 mM Tris, 2.5 mM $MgCl_2$, 12.5 U benzonase, 5 U CIAP, 0.15 U PDE I, and 5 ng coformycin; *THU+coformycin*: 5 mM Tris, 2.5 mM $MgCl_2$ 12.5 U benzonase, 5 U CIAP, 0.15 U PDE I, 5 ng coformycin, and 50 ng tetrahydrouridine (THU). **(B,C)** The impact of filters for ribonucleosides LCMS analysis. **(B)** Fold-change values for LC-MS/MS analysis of synthetic standards after filtration of tRNA hydrolysates with 3 commercial 10,000 Da filters: VWR (tube format), PALL (96-well plate format), and Agilent (96-well plate format). Fold-change was calculated relative to unfiltered signals. **(C)** Fold-change values for LC-MS/MS analysis of synthetic standards processed using a 0.2 µm inline filter. No significant column clogging was observed after ~1000 injections, despite the lack of enzyme removal by the 0.2 µm inline filter.

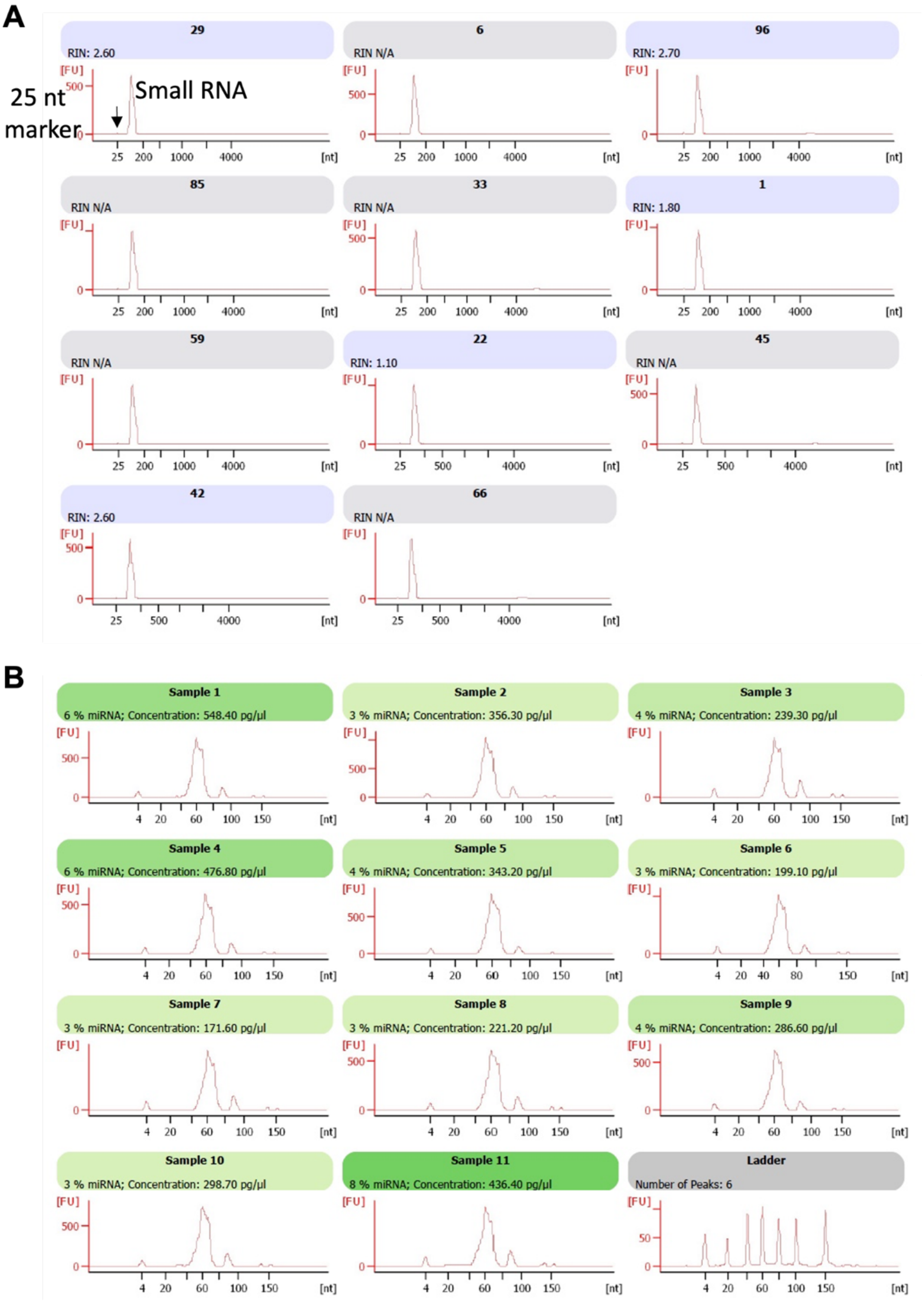

Supplementary Figure 7. Reproducibility of RNA purity for small RNA samples purified using the Tecan platform. Representative Bioanalyzer tracings for 96 small RNA samples purified from PA14 using **(A)** a pico chip and **(B)** a small RNA chip. The RNAs were overloaded to check potential rRNA contamination. All small RNA samples (mainly tRNA) were visibly free of large rRNA and consistently had small amounts of 5s rRNA, which is unlikely to bias modification analyses due to the apparent lack of modifications on 5S rRNA. The yield and purity of the 96 small RNA samples is shown in **Fig. 1B.**

A

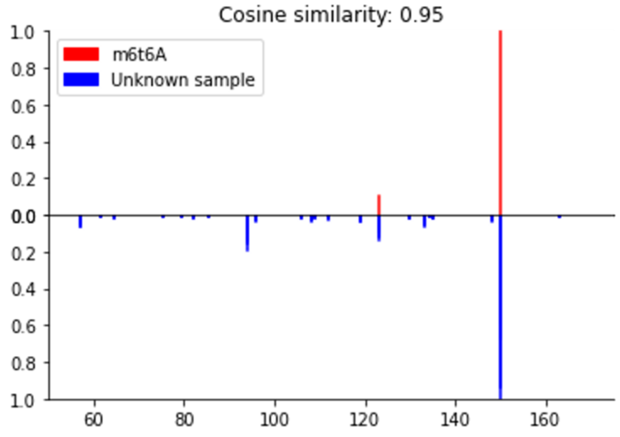

[M+H]⁺: 427.1577, found 427.1571, Δppm: -1.4

B

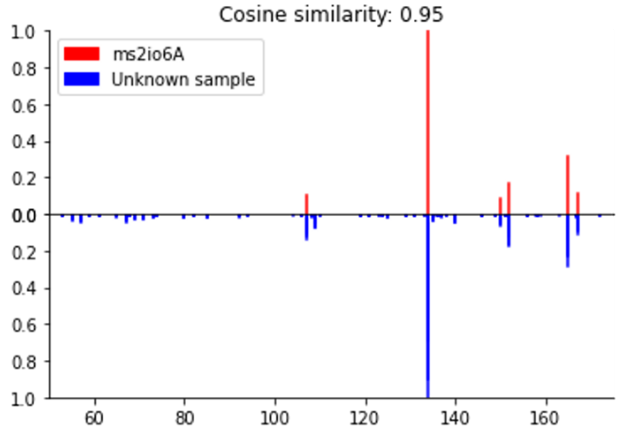

$[M+H]^+$: 398.1498, found 398.1494, Δppm: -1.0

Supplementary Figure 8. High-resolution mass spectrometry confirmation of signals (m^6t^6A and ms^2io^6A) found by neutral loss scan in PA14 WT strain. The signals of m^6t^6A (**A**) and ms^2io^6A (**B**) were confirmed by higher-energy collisional dissociation (HCD)-MS and spectral library matching by injecting 2 μg hydrolyzed tRNA. The LC and MS parameters strictly follow the protocol as previously reported(2). No U modifications were discovered using this method.

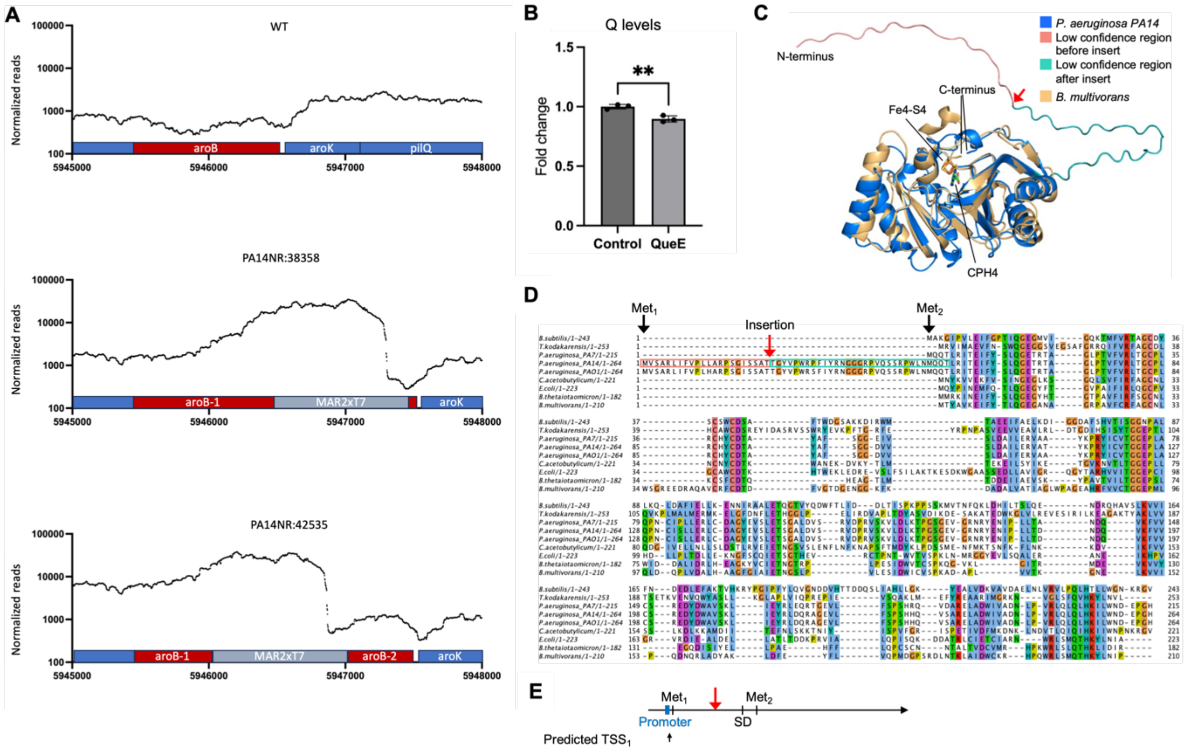

Supplementary Figure 9. Resolution of transposon mutagenesis artifacts in PA14. (A) Visualization of RNA-Seq coverage across the aroB region of PA14 WT, aroB mutant PA14NR:42535 and aroB mutant PA14NR:38358. Black curves represent read coverage, and the aroB gene is highlighted in red. The introduction of the high-expression transposon MAR2xT7 elevated expression of a truncated aroB upstream of this transposon. In PA14NR:42535, the truncated aroB has a 32bp deletion at the 5'end, potentially allowing for the synthesis of a functional N-truncated AroB protein. While in PA14NR:38358, the truncated aroB is too short to produce functional AroB protein. **(B-E)** Curation of the translation start site of QueE. **(B)** The Q levels of the QueE mutant compared to the control analyzed using LC-MS with three replicates. **(C)** Structure alignment of PA14 QueE (blue, red and green cartoon) and *B. multivorans* QueE protein (yellow cartoon, PDB 4njh). The iron-sulfur cluster and CPH4 (6-carboxy-5,6,7,8-tetrahydropterin) are represent in sticks. The structure of PA14 QueE was predicted by AlphaFold. The position of insertion of the transposon is indicated by the red arrow. The low confidence regions before and after the insertion of transposon were colored in red and green, respectively, and boxed accordingly in the multiple sequence alignment. **(D)** Multiple sequence alignment of QueE proteins. Two methionine are indicated by the black arrow. **(E)** The prediction of promoter, transcription start site (TSS) and Shine-Dalgarno (SD) sequence for *queE* gene.

A

Modification	Gene ID	Gene Name	Known function	Large RNA	Small RNA
C_m	PA14_68100*	trmS		0.944±0.062	0.735±0.024
	PA14_68110*			1.018±0.098	0.765±0.005
G_m	PA14_68100*	trmS		0.960±0.029	0.007±0.001
	PA14_68110*			0.971±0.040	0.292±0.036
	PA14_65190	rlmB	23s rRNA (2251)	0.055±0.063	1.057±0.042
m^2A	PA14_14830	rlmN	23s rRNA (2508) tRNA (37)	0.024±0.026	0.214±0.014
	PA14_40730*	trmV		0.991±0.034	0.841±0.027
m^6A	PA14_14340*	rlmF	23s rRNA (1618)	0.548±0.003	0.008±0.001
	PA14_66340	rlmJ	23s rRNA (2030)	0.467±0.001	1.047±0.041
m^6_2A	PA14_07730	rsmA	16s rRNA (1518/1519)	0.008±0.001	0.086±0.026

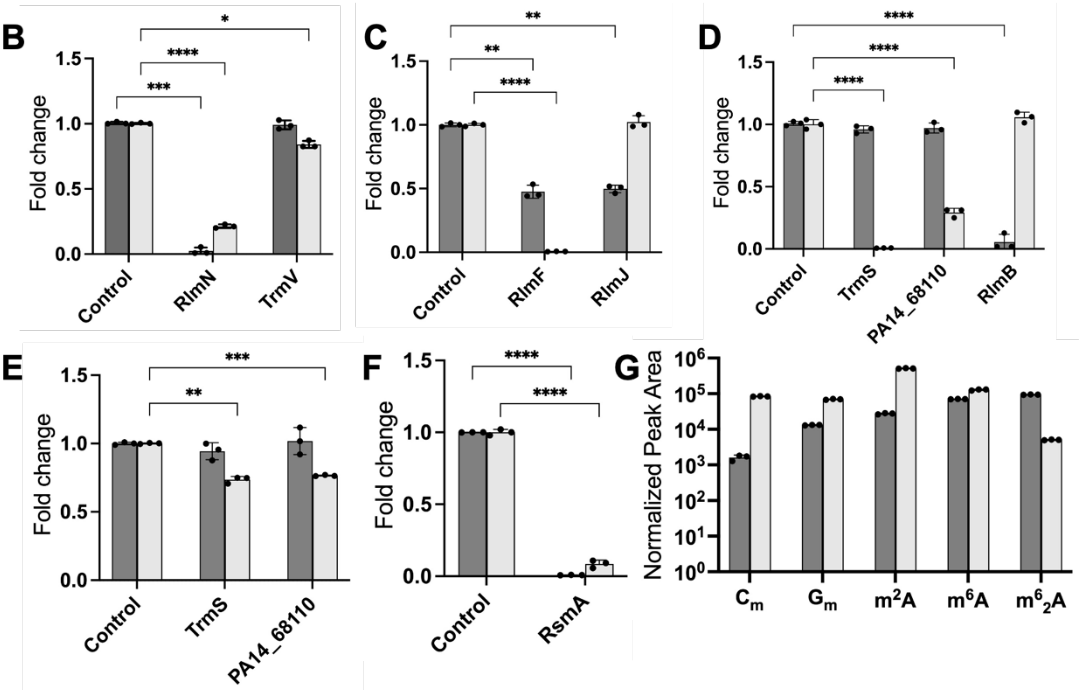

Supplementary Figure 10. Functional annotation of genes for RNA-modifying enzymes. (A) Summary of enzymatic functions annotated in this study in comparison to existing enzymes responsible for the same modifications. Asterisks denote newly annotated enzymes. Fold-change values for modifications were calculated relative to a transposon intergenic mutant control (n=3). **(B-G)** Analysis of modification levels in large RNA and small RNA fractions from PA14. Dark gray: large RNA; light gray: small RNA. **(B)** m^2A levels of PA14_14830 (RlmN) and PA14_40730 (TrmV) mutants. **(C)** m^6A levels in RlmF and RlmJ mutants. **(D)** Gm levels in PA14_68100, PA14_68110 and PA14_65190 (RlmB) mutants. **(E)** Cm levels in PA14_68100 (TrmS), PA14_68110 and PA14_65190 (RlmB) mutants. **(F)** $m^6{}_2A$ levels in PA14_07730 (RsmA) mutant. Fold-change values were calculated relative a transposon intergenic mutant control. **(G)** The relative abundance of modifications in large RNA and small RNA fractions. Note the $\log_{10}$ y-axis scale. (B-F) Statistical significance is indicated as follows: ns (not significant), * ($p < 0.05$), *** ($p < 0.001$), and **** ($p < 0.0001$), determined by *two-way ANOVA* test.

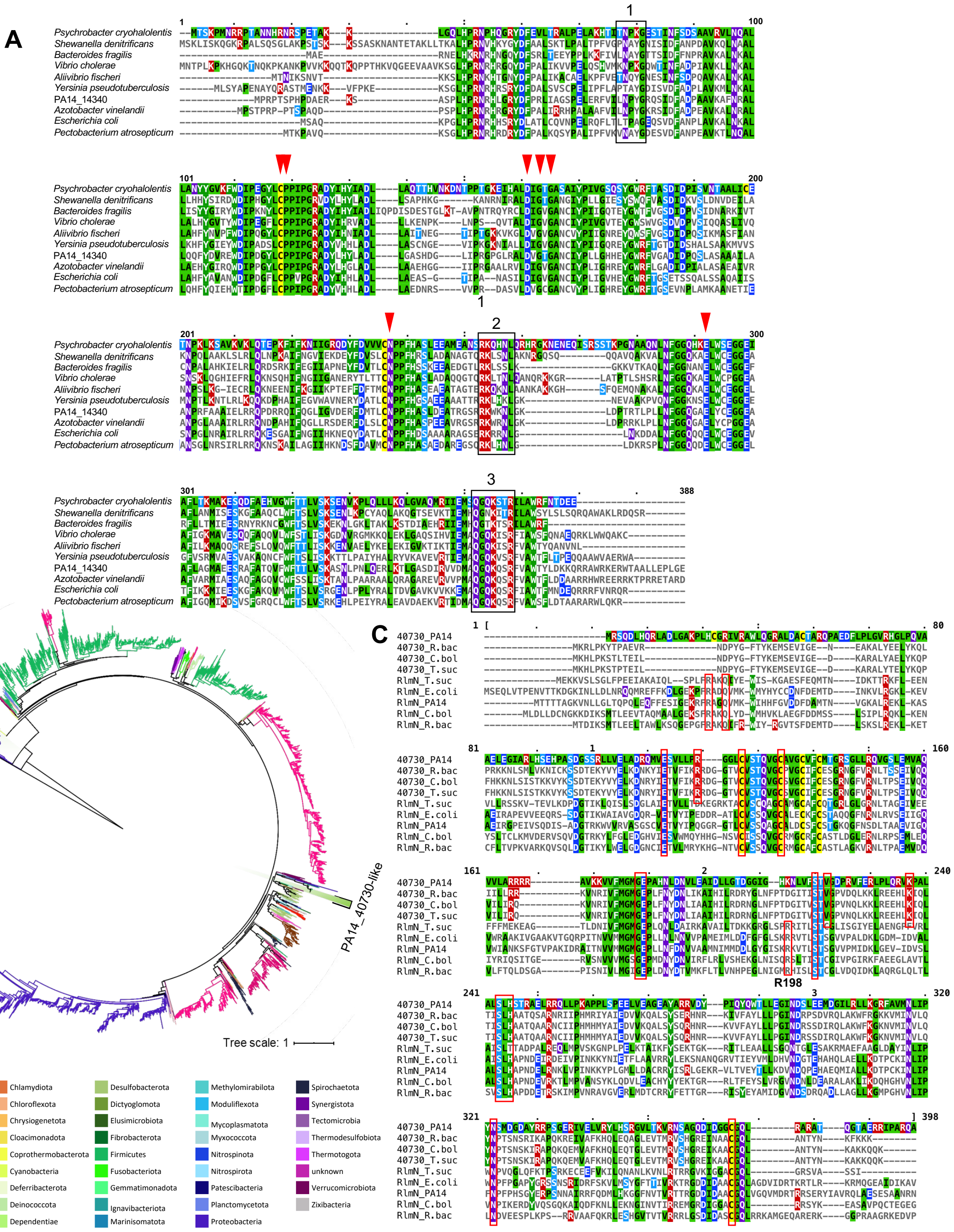

Supplementary Figure 11. Comparison of PA14_14340 and PA14_40730 with RlmF-like and RlmN-like proteins from other bacteria. (A) Sequence alignment of PA14_14340 and 9 RlmF proteins. Amino acid coloring reflects physicochemical properties. Red triangles: conserved residues proximal to SAH. Boxes: 3 turns predicted in PA14_14340 protein structure that fit the tRNA ASL groove. Dashes: sequence alignment gaps. Uniprot IDs are listed in **Supplementary Table 3**. **(B)** Maximum-likelihood phylogenetic tree of 5965 RlmN and 136 PA14_40730 like proteins. Sequences obtained by BLASTp search of 6616 bacterial genomes, with METTL3 proteins in human, mouse and drosophila as the out group. Branches are colored by phylogenetic affiliation at phylum level. Light green sections are PA14_40730-like proteins; others are RlmN homologs. **(C)** Sequence alignment of RlmN-like and 40730-like protein sequences. Amino acid coloring reflects physicochemical properties. Boxes: conserved residues within or across protein families. Dashes: gaps in the sequence alignment. Protein IDs used for alignment are listed in **Supplementary Table 3.**

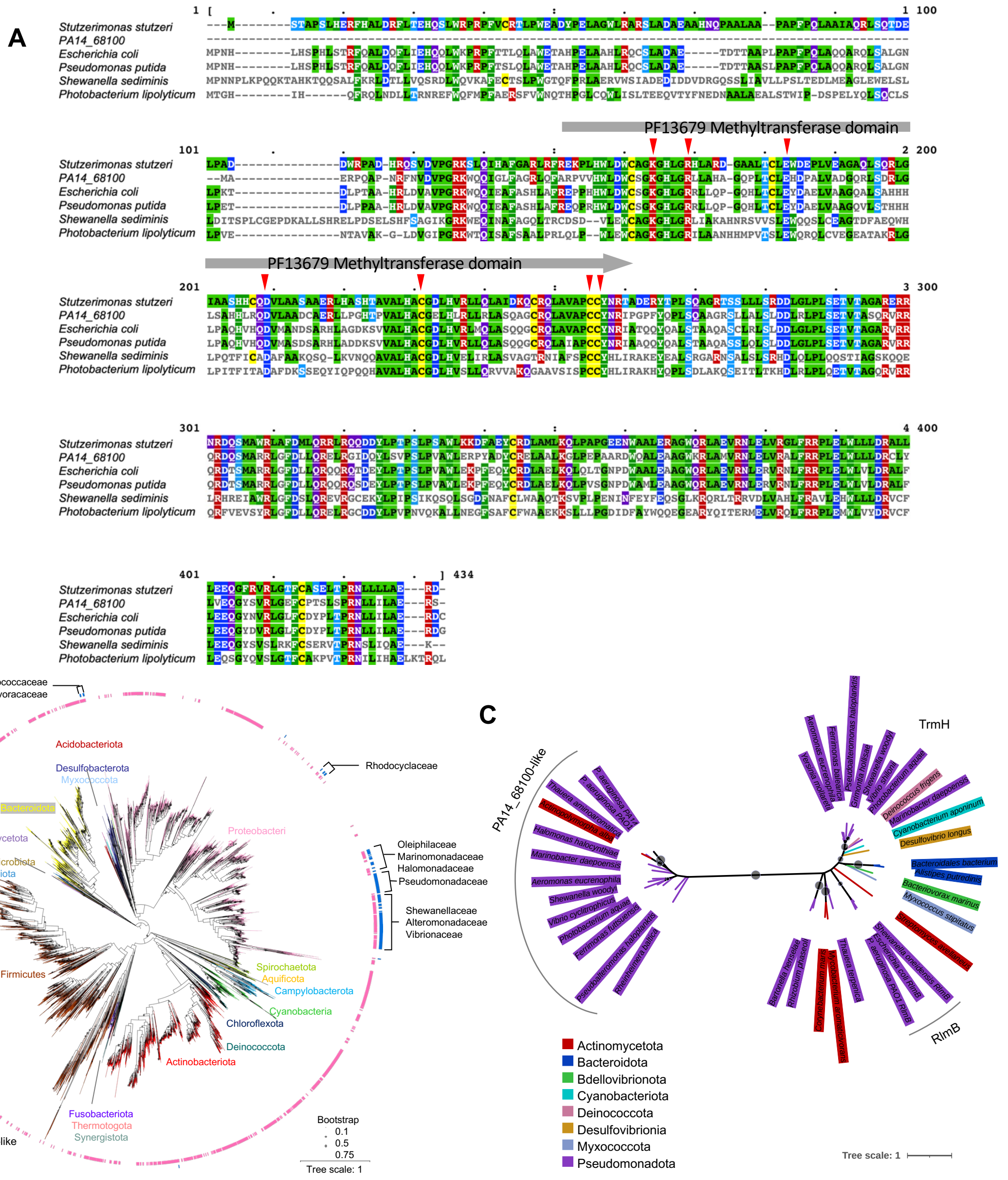

Supplementary Figure 12. PA14_68100 is a non-orthologous displacement of *E. coli* TrmH. (A) Sequence alignment of PA14_68100 homologs. Sequences were retrieved from Uniprot using BLASTp. Amino acid coloring reflects physicochemical properties. Conserved residues proximal to RNA and SAM substrates are noted by triangles. Protein IDs used for alignment were listed in Supplementary Table 8. **(B,C)** Taxonomic and phylogenetic analysis of TrmH and PA14_68100-like proteins. **(B)** Distribution of TrmH and PA14_68100-like proteins in bacterial genomes. A maximum likelihood tree of 10 concatenated ribosomal proteins was created for the 6,616 complete genomes in the BV-BRC database (https://www.bv-brc.org/). The branches are colored by phyla. *Outer circles*: TrmH (red) and PA14_68100-like proteins (blue). *Inner circle*: families encoding PA14_68100-like proteins. *Dots*: branches with bootstrap support value <0.75. For better visualization, phyla with few leaves are not annotated. **(C)** A maximum-likelihood phylogenetic tree of TrmH, RlmB and PA14_68100 like proteins from 6616 bacterial genomes. Branches are colored by phylogenetic affiliation at phylum level. Dots: Branches with bootsrap values <0.5.

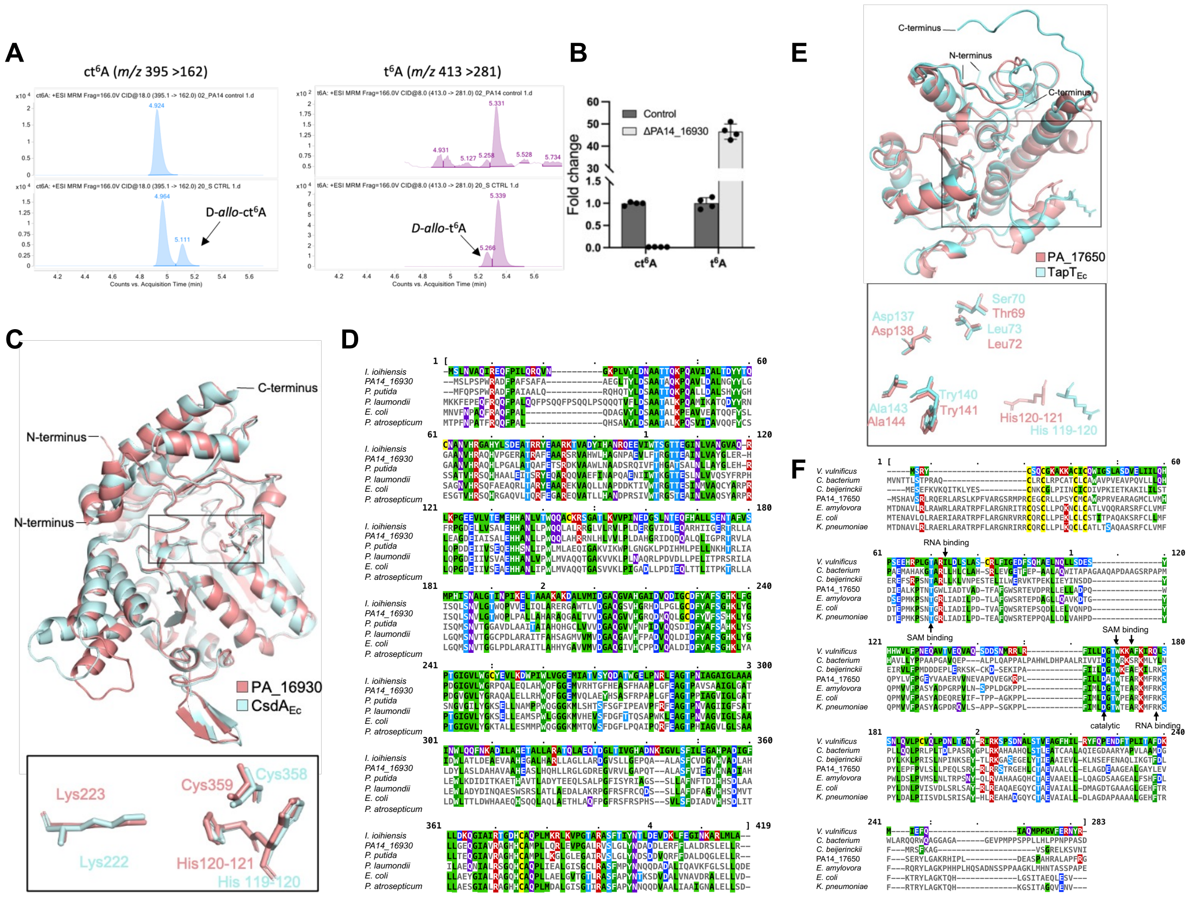

Supplementary Figure 13. Annotation of PA14_16930 as CsdA involved in ct^{6}A formation (A-D) and alignment of PA14_17650 and TapT$_{Ec}$ (E,F). **(A)** Identification of ct^{6}A and t^{6}A is complicated by RNA processing artifacts. Extracted ion chromatograms of ct^{6}A and t^{6}A in tRNA hydrolyzed in basic Tris-containing buffer (lower panel) and neutral Tris-free conditions. The isolation and hydrolysis of tRNA were performed under acidic and neutral Tris-free conditions following the protocol of Miyauchi *et al.*[2] **(B)** Analysis of ct^{6}A and t^{6}A levels in total RNA from the CsdA (PA14_16930) mutant strain using neutral RNA hydrolysis protocol. The fold-change values were calculated relative a transposon intergenic mutant control (n=4). **(C)** Alignment of predicted structure of PA14_16930 and crystal structure of CsdA in *E. coli* (PDB 5FT4). Three conserved residues in the sulfur transition are depicted in the box. **(D)** Sequence alignment of PA14_16930 and CsdA proteins. The conserved residues in sulfur transition are boxed. Uniport ID: *I. ioihiensis*, Idiomarina loihiensis, Q5QXG2, *P. putida*, *Pseudomonas putida*, Q9Z408, *P. laumondii*, *Photorhabdus laumondii*, Q7N8R7, *E. coli*, Q46925, *P. atrosepticum*, *Pectobacterium atrosepticum*, Q6D8G2. **(E)** The structural alignment of predicted structure of PA14_17650 and predicted structure of TapT in *E. coli* (Uniport Q47319). The stick cartoon represents the conserved residues in the sulfur transition (boxed on right). **(F)** The sequence alignment of PA14_17650 and TapT proteins. The conserved residues in substrate binding are indicated. UniprotID: *E. coli*, Q47319, *Erwinia amylovora*, D4HZ44, *Vibrio vulnificus*, Q7MMG0, *Klebsiella pneumoniae*, W1DMM7, *Clostridium beijerinckii*, Q549D3, *Comamonadaceae bacterium*, A0A4Q3M049.

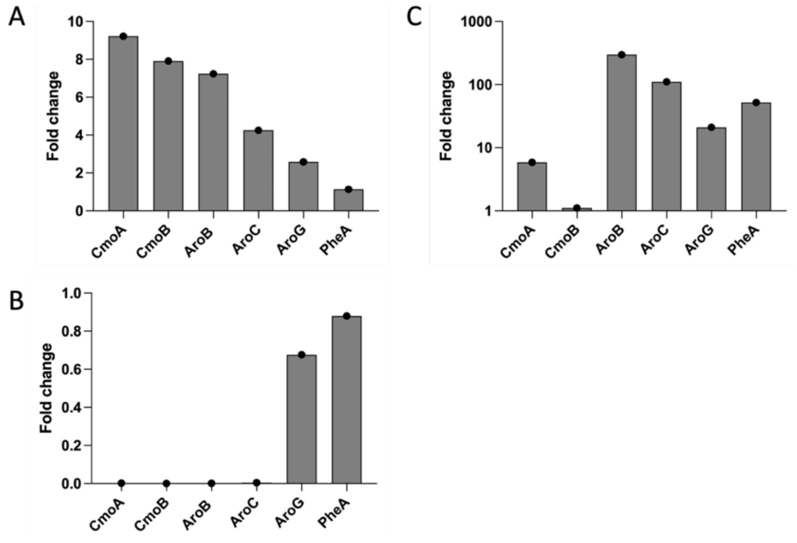

Supplementary Figure 14. Levels of ho^5U (A), cmo^5U (B) and mo^5U (C) in mutants involved in shikimate pathway. Fold-change values for ho^5U and cmo^5U in mutant PA14 strains are derived from **Supplementary Table 6,** with details in the **Methods** section. The fold-change value for mo^5U in mutants is relative to the noise level in samples in which mo^5U was not detected, analyzed on the same day as corresponding mutants to control for day-to-day instrumental variations.

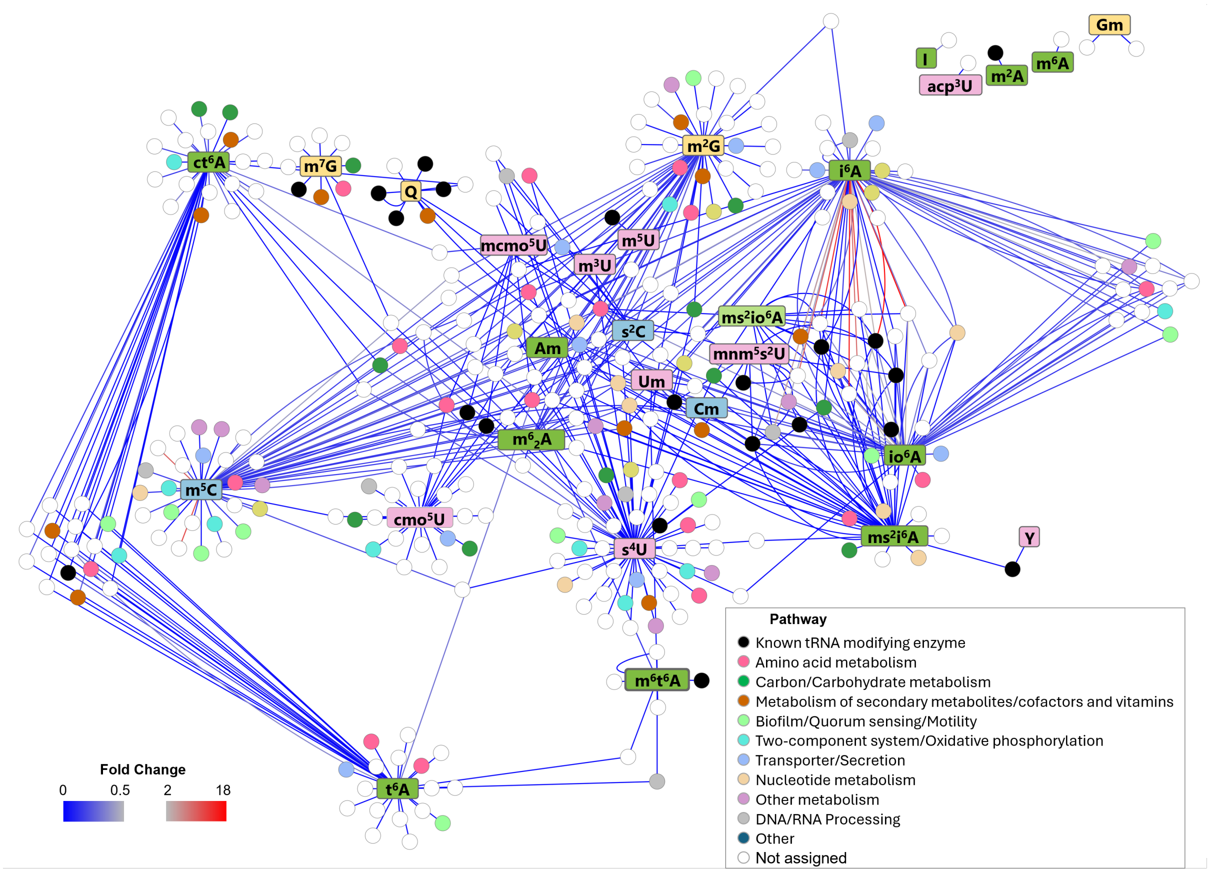

Supplementary Figure 15. Protein-modification network of 28 RNA modifications linked to 313 PA14 mutant genes (nodes/circles) that affect modification levels. The color of the edges (lines connecting nodes) indicates the modification fold-change (increasing red, decreasing blue; see lower left key) and the color of filled node/circle indicates the selected pathway of the gene as detailed in the pathway key lower right. The white/open circles are not assigned to any pathway. Modifications D and m1G are not shown here given the lack of change in these modifications in any PA14 mutants.

Supplementary Tables

Supplementary Table 1. Configuration of Tecan EVO150 parameters used for magnetic beads-based tRNA isolation from crude lysates. *The tips for wash buffer aliquot are re-cycled, so the dispense "move tips to left" function is not used to avoid potential contamination. #Tips for supernatant removal are also used for beads wash after tip wash, so the "move tips to left" function is not used in "Tip wash" step to avoid contamination in Waste plate. &Command "Beads wash buffer residue removal" was repeated twice, and the interval between each command is 1 min to ensure complete removal of ethanol.

	Aspirate parameters			Dispense parameters			
**Liquid transfer steps**	**Height (mm)**	**Speed (μL/s)**	**System trailing airgap (μL)**	**Height (mm)**	**Speed (μL/s)**	**Move tips to left (Y/N)**	**Repeat**
Cell lysate transfer	0.1	8	2	-0.5	15	Y	-
Cell lysate and Binding buffer-1 mix	-0.5	50	-	-1.5	20	Y	5
Wash buffer aliquot*	-0.5	45	-	-1	110	N	10
1st super-natant transfer	0	8	2	-0.5	15	Y	-
1st residue removal	0.1	8	3	1.5	15	Y	2
Tip wash#	-0.5	50	-	-1.5	200	N	-
Beads wash	-0.5	45	-	-1	110	Y	2
Wash buffer residue removal&	0.6	8	2	-0.5	15	Y	2
2nd super-natant transfer	-1.5	50	-	-1.5	20	Y	-
RNA elution	0.1	8	2	-0.2	40	Y	-
Supernatant discard	0.1	8	2	-1.5	20	Y	3

Supplementary Table 2. Dynamic MRM table for LC-MS/MS analysis of PA14 tRNA modifications. Ribonucleoside abbreviations follow the nomenclature for the "Short Name" in Modomics (http://genesilico.pl/modomics/modifications). # denotes a modification without a synthetic standard as reference.

Ribonucleoside Abbreviation	Precursor Ion	Product Ion	RT (min)	Collision Energy (eV)
^{15}N-dA	257	141	3.7	10
ac^{4}C	286	154	4.9	4
acp^{3}U	346	214	1.1	10
A$_{m}$	282	136	4.7	12
C$_{m}$	258	112	2	8
cmnm^{5}s^{2}U	348	141	1.9	16
cmnm^{5}U	332	200	0.8	8
cmo^{5}U	319	187	2.66	4
ct^{6}A$^{\#}$	395.1	162	5	18
D	247	115	0.7	5
G$_{m}$	298	152	4.6	8
gluQ$^{\#}$	539.2	407	uncertain	22
ho^{5}U	261	129	1	1
I	269	137	3.2	8
i^{6}A	336	204	6	12
io^{6}A	352.1	220	5.5	12
k^{2}C$^{\#}$	372.1	240.1	uncertain	5
m^{1}A	282	150	1	16
m^{1}G	298	166	4.6	8
m$^{2}_{2}$G	312	180	5.2	14
m^{2}A	282	150	5.1	16
m^{2}G	298	166	4.8	8
m^{3}C	258	126	1.1	8
m^{3}U	259	127	4.3	4
m^{4}C	258	126	0.94	8
m^{4}C$_{m}$$^{\#}$	272.1	126.1	uncertain	10
m^{5}C	258	126	1.3	2
m^{5}U	259	127	3.5	1
m$^{6}_{2}$A	296	164	5.4	20
m^{6}A	282	150	5.1	16
m^{6}A$_{m}$	296.1	150	5.4	12
m^{6}t^{6}A$^{\#}$	427	295	5.6	8
m^{7}G	298	166	1.7	6
m^{8}A$^{\#}$	282	150	uncertain	16
mcmo^{5}U	333	201	5.2	4
mnm^{5}s^{2}U	304	172	1.3	8
mnm^{5}U	288.1	156.1	1	8

mo^5U	275	143	3.8	4
ms^2i^6A	382.2	250.1	6.4	20
$ms^2io^6A^{\#}$	398	266	6	16
nm^5s^2U	290	158	0.9	4
nm^5U	274	152	0.75	8
$oQ\_quantifier^{\#}$	426	163	4	26
$oQ\_qualifier^{\#}$	426	295	4	8
$preQ_1$	312	163	1.7	14
Q	410	163	4.1	22
s^2C	260	128	1.4	8
s^2U	261	129	3.7	0
s^4U	261	129	4	0
$se^2U^{\#}$	307	175	uncertain	13
t^6A	413	281	5.4	8
Um	259	113	4.2	4
Y	245	191	0.7	20

Supplementary Table 4. Limit of detection (LOD) and limit of quantification (LOQ) of modified ribonucleosides using the rapid UHPLC/MS method. These modified ribonucleosides were detected in the presence of 1000-fold excess of canonical ribonucleosides (rA, rC, rU and rG) to account for the signal suppression effect during RNA sample analysis.

Modifications	Intercept	Std. Error (σ)	Slope (S)	R^2	LOD (fmol)	LOQ (fmol)
f^5C	87.54	193.2	2999	0.9996	0.19	0.64
hm^5C	72.89	67.86	658.9	0.9994	0.31	1.03
I	131.7	190.5	9599	0.9992	0.06	0.2
m^5C	64.33	137.5	916.4	0.9978	0.45	1.5
m^6A	217	76.39	2165	0.9974	0.11	0.35
m^6A_m	-28.93	83.92	3564	0.9989	0.07	0.24
mcm^5s^2U	-816.8	331.5	478.4	0.9995	2.08	6.93

Supplementary Results

Developing a high-throughput platform for tRNA modification profiling. The workflow for the HT tRNA analytical platform is shown in **Fig. 1A** and begins with growing cells (~1.6×10^8 CFU PA14, 5×10^5 HeLa cells) or placing tissue samples (10 mg) in wells of a 96-well plate. The samples are then subjected to cell lysis, removal of large RNA and genomic DNA, purification of small RNA, hydrolysis of RNA to ribonucleosides, LC-MS/MS analysis, and signal processing (**Fig. 1A**), with each step optimized as detailed in **Supplementary Figs 2-7**. Bacterial and human cells were all lysed using a buffer containing 4 M guanidine isothiocyanate (GITC) with shaking but without enzymatic or mechanical disruption. Animal tissues were lysed with the same buffer supplemented with mechanical disruption using a

tissue lyser (**Supplementary Fig. 3**). This method facilitated effective inhibition of RNases and other RNA-modifying enzyme activity and further enhanced the size selection resolution of magnetic beads for small RNA separation (**Supplementary Fig. 3**).

We next used a two-step magnetic beads-based method to isolate small RNA species from crude lysates in 96-well plate format (**Fig. 1A**). In the first step (1st Round), RNA binding buffer I containing magnetic beads, salts, and molecular crowding agent (PEG 8000) was added to capture genomic DNA and large RNAs (>150 nt, mainly rRNA and mRNA), leaving small RNAs (<150 nt, mainly tRNA) in the supernatant. In the second step (2nd Round), RNA binding buffer II containing isopropanol and fresh beads was added to the supernatant to capture small RNAs. Beads from both rounds 1 and 2 were washed and the nucleic acids eluted as described in **Methods**. This protocol was optimized for the composition of RNA binding buffers I and II (salts, pH, crowding reagents; **Supplementary Fig. 3C,D,E**) to maximize the yield and purity of the small RNAs. In our experiments, both silica- and carboxyl-coated magnetic beads were effective for bacterial and mammalian cells, but we settled on carboxyl-coated beads due to their compatibility with tissues (**Supplementary Fig. 3F**). The method proved to be as effective as silica column-based commercial kits in size resolution and tRNA yield (**Supplementary Fig. 3E**).

The cell lysis and tRNA purification steps were then adapted to a 96-well plate format and automated using a robotic liquid handler (Tecan EVO150). The Tecan workflow includes 9 steps for the tRNA purification process in 1 h for 96 samples (**Supplementary Fig. 2A**). To ensure consistent and robust results (**Fig. 1B, Supplementary Fig. 3**), we empirically determined the optimal labware and parameters for aspiration, dispensing, and mixing (**Supplementary Table 1**). For instance, the “Wash buffer residue” step was optimized and conducted twice to thoroughly eliminate ethanol residue, which addressed the issue of peak shape broadening during LC-MS/MS analysis (**Supplementary Fig. 2C**). Another important issue was removal of rRNA contamination as carryover from the 1st round of beads. We introduced a plate centrifugation action between the two magnet pull-downs to ensure efficient removal of 1st round beads (**Supplementary Fig. 2D**). Using the automated approach, the average yield of small RNA from approximately 0.3 OD_{600} *Pseudomonas* cells is 747±42 ng (n=96) with an average 260/280 nm ratio of 1.99±0.12 (**Fig. 1B**). Collectively, this method provided high-quality tRNA samples at low cost ($0.3 per sample) and high efficiency (1 h for 96 samples).

The second portion of the platform, LC-MS/MS analysis of RNA modifications, required optimization of both the HPLC resolution and the MS/MS quantification of ribonucleosides to increase sensitivity and reduce the typical run time of 20-30 minutes for one sample.(3) Here we coupled a rapid UHPLC method with dynamic multiple reaction monitoring for analysis of >60 RNA modifications in a 6-minute HPLC run (**Fig. 1A**). This approach was found to be applicable for analyses of tRNA from bacteria, mammalian cells, and animal tissues (**Supplementary Fig. 3**). Of the two compact columns assessed — BEH C18 and HSS T3 — the former outperformed the latter with a higher plate count and a shorter run time (**Supplementary Fig. 4**). Both the LC configurations (**Supplementary Fig. 4**) and MS parameters (**Supplementary Table 2**) were fine-tuned to optimize identification and quantification of modifications, including isobaric methylation isomers (**Supplementary Fig. 4**). The sensitivity of the method was confirmed with limits of detection and quantification for modified ribonucleosides in the low femtomole range, including problematic U modifications (**Supplementary Table 4**). Method performance was further assessed using tRNA from wild-type PA14, focusing on retention time stability, chromatographic peak characteristics, and signal carryover. The standard deviation for retention times was less than 5 seconds (**Supplementary Fig. 5A**), with coefficient of variation for peak area less than 10% (**Supplementary Fig. 5C**). The full width at half maximum (FWHM) for most ribonucleosides stayed under 5 seconds, only cmo^{5}U extended to 7 seconds (**Supplementary Fig. 5B**). No negligible sample carryover was observed throughout the spectrum (**Supplementary Fig.

5D,E). To validate the reliability of the rapid LC-MS/MS method, we analysed the same sample matrix (n=16) using both fast (6-minute) and conventional methods and found them to be strongly correlated (**Supplementary Fig. 5F**; Pearson correlation r = 0.927, $p < 0.0001$). In addition, the composition of enzyme cocktails for tRNA hydrolysis was examined based on previous work in our lab (**Supplementary Fig. 6**)(4). An adenosine deaminase inhibitor, here coformycin, was found to be essential to obtain accurate inosine levels, with deaminase contamination of enzyme preparations evident from increased inosine in the absence of coformycin. However, the cytidine deaminase inhibitor appears unnecessary as no significant m^3C/m^5C to m^3U/m^5U deamination was detected. Moreover, the inclusion of the iron chelator deferoxamine was proved to protect ho^5U from iron-induced Fenton reaction(4). Lastly, we replaced the polyethersulfone 10K spin-filter used in previous protocols with a 0.2 μM stainless steel inline filter to prevent significant loss of hydrophobic ribonucleosides (**Supplementary Fig. 6B,C**).

Finally, the third leg of the platform involves data processing pipeline to manage conversion of hardware-specific signals to normalized signal intensities comparable across different analytical runs, to collate signal intensities with gene names, and to calculate fold-change values relative to the adjusted mean of samples run in 2 h. This mean is calculated based on the expectation that the majority of mutants exhibit insignificant modification level changes. This is substantiated by our screening data, which indicates that over 94% of the 17,2860 measurements display less than 1.2-fold changes. This approach waives the comparison with wild-type strain which is suboptimal served as a control as it cannot be cultured in the same condition/plate as mutants. Additionally, the potential for signal drift is significant(5), given that LC-MS analysis of a single 96-well plate may require a full day. To mitigate these issues, we use an approach to calculate adjusted mean for fold change calculation. We initially calculated modification levels by averaging UV-normalized peak areas for each row. Then, we eliminated any data points exhibiting more than a two-fold change before recalculating the final average, which then served as the baseline for fold change calculations for each respective row. The details of the data processing are described in **Methods**.

The fully optimized platform allows processing of one 96-well plate per hour for tRNA purification and hydrolysis, followed by one plate processed every 15 hours for LC-MS/MS analyses of 96 samples (9.4 min per sample). The tRNA modifications analytical platform was now applied to analyze the effects of 4,600 gene products on the levels of 41 tRNA modifications in *P. aeruginosa* PA14.

tRNA modification profiling of a PA14 transposon insertion mutant library: Resolving discrepancies. As with any transposon insertion library, some care must be taken in interpreting the results. For example, loss of putative *uspA* (*PA14_41440*) increased io^6A and the io^6A/i^6A ratio (**Supplementary Table 6**), suggesting that *miaE* expression or activity was upregulated (see **Fig. 3C**). In the PA14 genome, *miaE* (*PA14_41430*) shares a promoter region with *uspA* in a 'head-to-head' orientation. One potential explanation for elevated MiaE levels in the *uspA* knockout is that accumulated stress in *uspA* mutant stimulates an alternative sigma factor to bind to the *uspA* promoter region, causing upregulation of *miaE* as a 'side effect'. Another potential reason is that insertion of a transposon has obvious polar effects on the expression of downstream genes but can also affect the expression of upstream genes(6). In this case, *miaE* is located ~700 bp upstream of a transposon insertion site and its expression is upregulated. In two other cases, reduced tRNA modification levels are observed. For the *yajC* mutant, the insertion site was ~200 bp downstream of the *tgt* gene, which could cause the observed reduction in Q (**Supplementary Table 6**), while the insertion site for the *thiG* mutant was ~1000 bp upstream of the *trmB* gene, possibly causing the reduction of m^7G (**Supplementary Table 6**). DNA sequencing analysis of several mutants was performed to confirm strain identities, revealing that several mutants differed from the library listings. The results have been summarized in **Supplementary Table 8**. For example, the transposon insertion site in the *trmD* (*PA14_15990*) mutant was located at an intergenic region, thus no

reduction of m^1G level was observed. As another example, repeated LC-MS/MS analyses of the two *aroB* knockouts PA14NR:38358 and PA14NR:42535 consistently showed cmo^5U absent in PA14NR:38358 but present in PA14NR:42535. mRNA sequencing indicated that the regions upstream of the transposon insertion site are expressed at 10-fold higher levels than wild-type. For PA14NR:42535, the insertion site is only 32 bp from the translation initial site of *aroB* (**Supplementary Fig. 9A**). The resulting truncated *aroB* mRNA could produce a functional N-truncated AroB protein using an alternative translation start site, with 10-fold increased expression offsetting the reduced activity of the truncated AroB protein. A similar scenario might also be occurring in the mutant PA14NR:29841, where the transposon is inserted into the 5' end of the *pdxA* gene, thus PdxA protein is still functional and no noticeable level change in corresponding RNA modifications as seen in another mutant PA14NR:40435 (**Supplementary Table 6,8**). In another case, the deletion of *PA14_51680* (*queE*) resulted in only 10% reduction of Q (**Supplementary Table 6, Supplementary Fig. 9B**). The transposon was inserted between two methionine residues in the N-terminus, the structure of which was difficult to model (**Supplementary Fig. 9C,D**). Taken together with the predicted promoter and Shine-Dalgarno (SD) sequence (**Supplementary Fig. 9E**), our findings suggested that the transcription of *queE* was disturbed in the mutant and that the second methionine is the initiator methionine unlike the original annotation.

Supplementary References

1. Sarid, L., Sun, J., Chittrakanwong, J., Trebicz-Geffen, M., Ye, J., Dedon, P.C. and Ankri, S. (2022) Queuine Salvaging in the Human Parasite Entamoeba histolytica. *Cells*, **11**.
2. Jora, M., Corcoran, D., Parungao, G.G., Lobue, P.A., Oliveira, L.F.L., Stan, G., Addepalli, B. and Limbach, P.A. (2022) Higher-Energy Collisional Dissociation Mass Spectral Networks for the Rapid, Semi-automated Characterization of Known and Unknown Ribonucleoside Modifications. *Anal Chem*, **94**, 13958-13967.
3. Su, D., Chan, C.T., Gu, C., Lim, K.S., Chionh, Y.H., McBee, M.E., Russell, B.S., Babu, I.R., Begley, T.J. and Dedon, P.C. (2014) Quantitative analysis of ribonucleoside modifications in tRNA by HPLC-coupled mass spectrometry. *Nat Protoc*, **9**, 828-841.
4. Cai, W.M., Chionh, Y.H., Hia, F., Gu, C., Kellner, S., McBee, M.E., Ng, C.S., Pang, Y.L.J., Prestwich, E.G., Lim, K.S. *et al.* (2015) In He, C. (ed.), *Methods in Enzymology*. Academic Press, Vol. 560, pp. 29-71.
5. Jiang, F., Liu, Q., Li, Q., Zhang, S., Qu, X., Zhu, J., Zhong, G. and Huang, M. (2020) Signal Drift in Liquid Chromatography Tandem Mass Spectrometry and Its Internal Standard Calibration Strategy for Quantitative Analysis. *Anal Chem*, **92**, 7690-7698.
6. Egorov, A.A., Alexandrov, A.I., Urakov, Valery N., Makeeva, Desislava S., Edakin, Roman O., Kushchenko, A.S., Gladyshev, Vadim N., Kulakovskiy, Ivan V. and Dmitriev, Sergey E. (2021) A standard knockout procedure alters expression of adjacent loci at the translational level. *Nucleic Acids Research*, **49**, 11134-11144.
